# Combined assessment of the TNM stage and *BRAF* mutational status at diagnosis in sporadic colorectal cancer patients

**DOI:** 10.18632/oncotarget.25300

**Published:** 2018-05-08

**Authors:** José María Sayagués, Sofía Del Carmen, María Del Mar Abad, Luís Antonio Corchete, Oscar Bengoechea, María Fernanda Anduaga, María Jesús Baldeón, Juan Jesús Cruz, Jose Antonio Alcazar, María Angoso, Marcos González, Jacinto García, Luís Muñoz-Bellvis, Alberto Orfao, María Eugenia Sarasquete

**Affiliations:** ^1^ Cytometry Service-NUCLEUS, Department of Medicine, Cancer Research Center (IBMCC-CSIC/USAL), CIBERONC and IBSAL (University Hospital of Salamanca), Salamanca, Spain; ^2^ Department of Pathology, University Hospital of Salamanca, Salamanca, Spain; ^3^ Cáncer Research Center and Service of Hematology (University Hospital of Salamanca), Salamanca, Spain; ^4^ Service of General and Gastrointestinal Surgery, University Hospital of Salamanca, Salamanca, Spain; ^5^ Department of Oncology (University Hospital of Salamanca) and IBSAL (University Hospital of Salamanca), Salamanca, Spain

**Keywords:** colorectal cancer, anti-EGFR therapy, BRAF V600E mutation, prognosis

## Abstract

The prognostic impact of *KRAS* mutations and other *KRAS*-related and non-related genes such as *BRAF, NRAS* and *TP53*, on sporadic colorectal cancer (sCRC) remain controversial and/or have not been fully established. Here we investigated the frequency of such mutations in primary sCRC tumors and their impact on patient progression-free survival (PFS) and overall survival (OS). Primary tumor tissues from 87 sCRC patients were analysed using a custom-built next generation sequencing (NGS) panel to assess the hotspot mutated regions of *KRAS/NRAS* (exons 2, 3 and 4), *BRAF* (exon 15) and *TP53* (all exons). Overall, mutations in these genes were detected in 46/87 sCRC tumors analyzed (53%) with the following frequencies per gene: *TP53*, 33%; *KRAS*, 28%; *BRAF*, 7%; and *NRAS*, 1%. A significant association was found between *KRAS* mutations and right side colon tumor location (p=0.05), well-differentiated tumors (p=0.04) and absence of lymphovascular invasion (p=0.05). In turn, *BRAF*-mutated tumors frequently corresponded to poorly- or moderately-differentiated sCRC (p=0.02) and showed a higher frequency of peritoneal carcinomatosis (p=0.006) and microsatellite instability (p=0.007). From the prognostic point of view, the *BRAF* mutational status together with the TNM stage were the only variables that showed an independent adverse impact on patient outcome in the multivariate analyses for both PFS and OS. Based on these results a scoring system was built and patients were classified into three prognostic subgroups with different PFS rates at 2 years: 91% vs. 77% vs. 0%, respectively (p<0.0001). Additional prospective studies in larger series of sCRC patients where mutations in genes other than those investigated here are required to validate the utility of the proposed predictive model.

## INTRODUCTION

Sporadic colorectal cancer (sCRC) ranks the third most common type of cancer worldwide, both in men and women, and the fourth leading cause of cancer-related death [1, 2]. In the last decades, the introduction of targeted therapies has led to great progress in tumor response to treatment and patient survival in many cancer types, including sCRC, but only selected subgroups of patients within a given tumor type may benefit from these novel agents (e.g. anti-EGFR and anti-VEGF therapies) based on better responses to therapy and a significantly improved outcome [3]. At present, the prognostic impact of KRAS mutations, and of mutations in other KRAS-related and unrelated genes such as NRAS and TP53 frequently mutated in sCRC, remains controversial, particularly as regards their impact on patient progression-free survival (PFS) and overall survival (OS) [4–6]. Despite this, the presence of KRAS and NRAS gene mutations, currently precludes administration of anti-EGFR directed therapies in sCRC patients; this translating into a lack of benefit of such novel therapies in this large subgroup of sCRC patients [7]. In turn, preliminary data suggests that the activating BRAF V600E mutation that occurs in a smaller fraction (5-10%) of sCRC patients who lack KRAS mutations, might be associated with a worse patient outcome [8] and significantly shorter OS rates. [9, 10]. Similarly, NRAS mutations occur in only a small fraction (1-6%) of all colorectal tumors [11] and they have been associated with reduced response to monoclonal antibody therapies [12], its potential prognostic value among early stage tumors, still requiring further confirmation in larger series of unselected sCRC patients. Finally, despite TP53 mutations are frequently detected in colorectal tumors (∼40% of cases), their specific prognostic impact on the outcome and survival of sCRC patients, still remains controversial.

Since 2013, the European Medicines Agency (EMEA) requires that exons 2-3-4 of both KRAS and NRAS are investigated prior to usage of novel targeted (e.g. anti-EGFR) therapies. From the onward, the number of molecular targets required to be analyzed in sCRC tumors has further expanded and it is expected to increase even more in the near future. Because of this (and other reasons) Sanger sequencing and other traditional sequencing approaches that interrogate the tumor DNA for specific variants of one (or a few) genes are being progressively replaced by next-generation sequencing (NGS). NGS allows simultaneous analysis of multiple gene targets with higher sensitivity, and at lower cost, using reduced amounts of DNA [13]. Thus, an NGS-based approach for simultaneous evaluation of KRAS, NRAS, BRAF, and TP53 gene mutations in hot-spot regions suitable for implementation into routine diagnostics in sCRC patients would be mostly welcome. Other genes that are recurrently mutated in sCRC and that have been associated to tumor response to therapy and/or the outcome of sCRC patients such as the SMAD4 and PIK3CA genes present in ∼10-35% and 10%-20% of cases, respectively [14–16], were not considered in the gene panel built for this study.

In the present study, we designed an NGS-based approach for simultaneous identification of hotspot KRAS, NRAS, BRAF and TP53 gene mutations in 87 primary sCRC tumors and evaluated the prognostic impact of the mutations identified in patient PFS and OS. Overall, the BRAF V600E mutation emerged as an independent prognostic factor for both PFS and OS, together with the TNM stage; based on the combined assessment of both parameters at diagnosis a prognostic score was built for stratification of sCRC patients into 3 risk groups with significantly different PFS rates.

## RESULTS

### Patient characteristics

Overall, 87 patients diagnosed with sCRC at the HUS (51 males and 36 females; median age of 72 years, ranging from 38 to 91 years) were studied. Median follow-up at the moment of closing the study was 19 months (range: 8 to 36 months). According to the histological type, 80 cases corresponded to adenocarcinomas, 4 cases to signet ring cell carcinomas, 2 cases to mucinous adenocarcinomas and one case to a neuroendocrine tumor. According to tumor grade, 24 cases were classified as well-differentiated tumors, 49 as moderately- and 7 as poorly-differentiated carcinomas. In all cases, histopathological grade was systematically confirmed in a second independent evaluation by another experienced pathologist. The most relevant clinical and laboratory data for each individual sCRC patient studied are summarized in Table 1.

**Table 1:** Clinical and biological characteristics of sporadic colorectal cancer (sCRC) patients analyzed in this study (n=87)

Disease features	Distribution
Age (years)^*^	72 (38-91)
**Gender**	
Female	36 (41%)
Male	51 (59%)
**Site of primary tumor**	
Right colon	44 (51%)
Left colon	39 (44%)
Rectum	4 (5%)
**Histological type**	
Adenocarcinoma	80 (92%)
Signet ring cell carcinoma	4 (5%)
Mucinous adenocarcinoma	2 (2%)
Neuroendocrine tumor	1 (1%)
**Grade of differentiation**^δ^	
Well-differentiated	24 (30%)
Moderate-differentiated	49 (61%)
Poorly-differentiated	7 (9%)
**Histopathologic tumor classification**	
pTis	3 (3%)
pT1	3 (3%)
pT2	20 (23%)
pT3	48 (56%)
pT4a	10 (12%)
pT4b	3 (3%)
**Histopathologic lymph node status**	
pN0	46 (53%)
pN1	25 (29%)
pN2	16 (18%)
**Metastatic status**	
M0	68 (78%)
M1	19 (22%)
**TNM stage at diagnosis**	
Stage 0	3 (3%)
Stage I	16 (19%)
Stage II	27 (31%)
Stage III	35 (40%)
Stage IV	6 (7%)
Tumor size (cm)^*^	4 (0.3-13)
CEA serum levels^*^	3.5 (0.5-35)
≤5 ng/ml	47 (62%)
>5 ng/ml	29 (38%)
**Lymphovascular invasion**	
No	68 (78%)
Yes	19 (22%)
**Perineural invasion**	
No	65 (75%)
Yes	22 (25%)
**Adjuvant treatment**	
No	43 (61%)
Yes	34 (39%)
**Local recurrence**	
No	69 (79%)
Yes	18 (21%)
OS (months)^*^	19 (3-37)

Results expressed as number of cases (percentage) or ^*^as median (range). ^δ^In 7 patients the grade of differentiation was not determined because of a histological type not corresponding to an adenocarcinoma: 2 signet ring cell carcinomas, 4 mucinous colorectal adenocarcinomas and one neuroendocrine tumor; CEA: carcinoembryogenic antigen; OS: overall survival.

Eighty-five patients underwent complete tumor resection (R0), while the remaining two cases showed positive tumor tissue margins to be affected (one patient showed microscopical and one macroscopical involvement). Adjuvant treatment was administered to 34/87 cases (39%) including: Xelox in 17/34 patients (50%), Capecitabine in 12 cases (35 %), Tomox in another 2 (6%), Tomudex in 1 case (3%), Utefox in another patient (3%) and cisplatin etoposide in the remaining case (3%).

### NGS quality control

The custom NGS panel here designed, successfully amplified the 53 targeted amplicons, (covering the corresponding 5.3kb) with a mean depth of 2400x reads in all tumor samples analyzed (Supplementary Table 1). The reference sample, which included several mutations interrogated by our custom panel –e.g. *KRAS* (G12D and G13D), *NRAS* (Q61K) and *BRAF* (V600E)- was also successfully analyzed, the variant allele frequencies (VAF) detected by NGS being very similar to the expected VAF as estimated by the DNA input (Table 2). According to these results, our panel allows detection of variants at low VAF as confirmed by the detection of *KRAS* G12D variant (6% VAF).

**Table 2:** Variant allele frequencies (VAF) observed for the most common *BRAF* (V600E), *KRAS* (G12D, G13D) and *NRAS* (Q61K) mutations identified in the reference sample (Quantitative Multiplex DNA reference standard (Horizon Discovery, Cambridge, UK)) used in this study by our NGS approach

Variant alleles detected by NGS					
Gene	Mutation	Altered read depth	Total Read depth	VAF detected by NGS	Expected VAF
***BRAF***	V600E	600	6607	9.1%	10.5%
***KRAS***	G13D	269	1733	15%	15%
***KRAS***	G12D	175	1854	9.4%	6%
***NRAS***	Q61K	141	829	17%	12%

NGS: next generation sequencing; VAF: variant allele frequency.

Expected VAF (last column) was estimated according to the manufacturer information based on the number of copies of the mutation per microliter and the input of reference sample added to the reaction.

### Frequency and type of *KRAS*, *NRAS*, *BRAF* and *TP53* mutations detected

**Figure 1:**
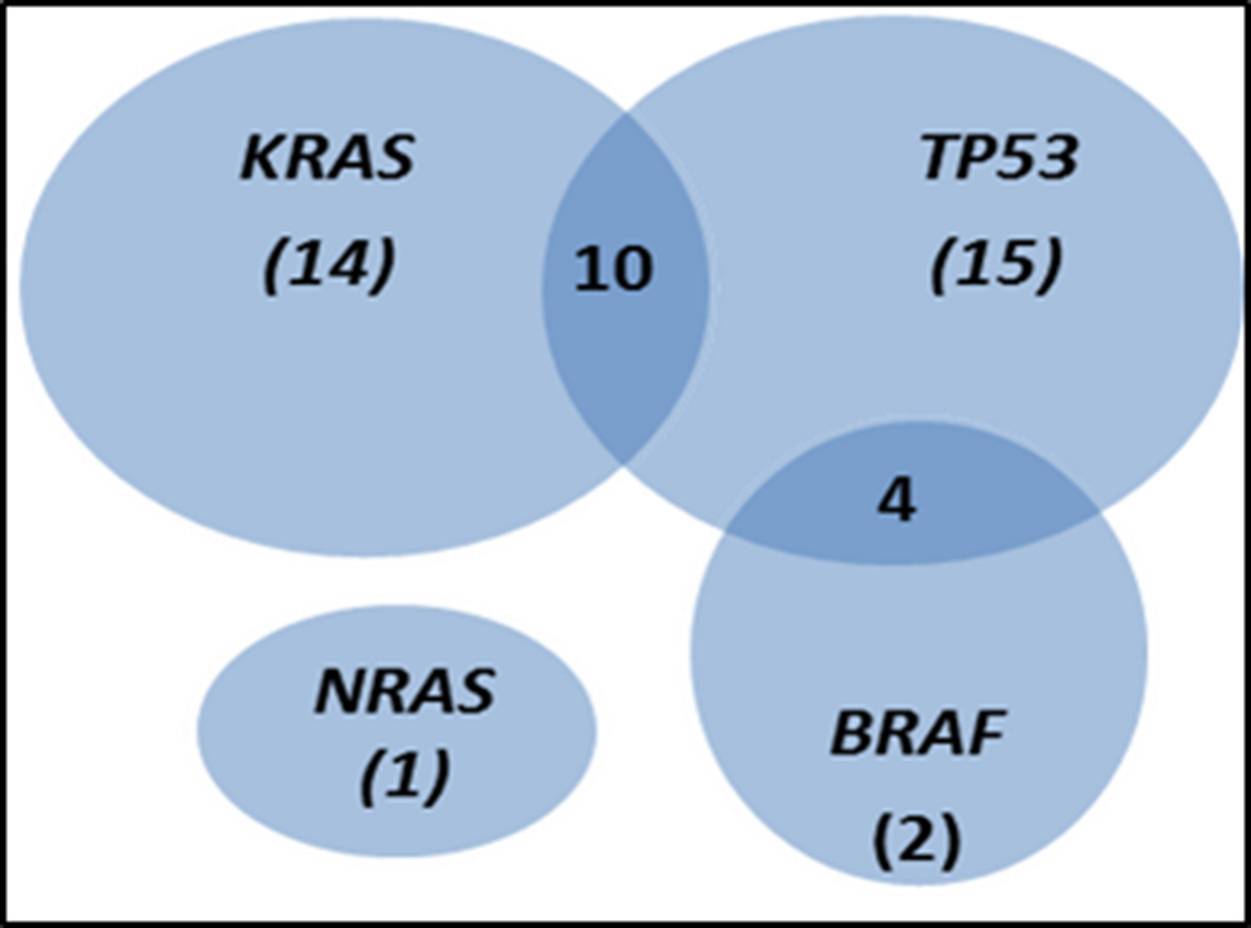
Venn diagram showing the distribution of *KRAS*, *NRAS*, *TP53* and *BRAF* mutations in the 87 sporadic colorectal cancer (sCRC) patients analysed in this study The number of mutated cases for each mutational profile is shown between brackets.

Overall, mutations were detected in 46/87 cases analyzed (53%). The *TP53* gene was the most frequently mutated gene (29/87 tumors; 33%), followed by the *KRAS* (mutated in 24/87 tumors; 28%) and *BRAF* (6/87 tumors; 7%) genes, while the *NRAS* gene was mutated in only one case (1%). The specific VAF for each individual mutation identified is listed in Supplementary Table 2. Regarding the individual hotspot mutations identified, *KRAS* G12D (8% of cases) and G12V (6%) corresponded to those showing the highest frequencies, followed by *BRAF* V600E (7%) and the *TP53* R282W (3%) and R175H (3%) variants (Table 3). Thirty-two cases (37%) showed one single mutation (the most common being *KRAS*), while 14 cases (16%) had two genes affected. Coexisting *TP53* and *BRAF* mutations were present in four cases (5%) whereas *TP53* and *KRAS* were simultaneously mutated in 10 (11%) patients (Figure 1). As expected, no single case carried (simultaneously) *KRAS* and *BRAF* mutations (Figure 1).

**Table 3:** Overall distribution of the *KRAS*, *NRAS*, *BRAF* and *TP53* gene mutations detected by next generation sequencing in the 87 colorectal cancer patients analyzed

Gene status	N. of cases (%)	Exon location	Mutation (N. of cases)
***KRAS***			
Wild type	63 (72%)		
Mutated	24 (28%)		
		Exon 2	G12D(7), G12V(5), G12A(4), G12S(1), G13D(2)
		Exon 3	Q61H(1), Q61L(1)
		Exon 4	A146P(1), A146T(1), K147E(1)
***NRAS***			
Wild type	86 (99%)		
Mutated	1 (1%)		
		Exon 3	Q61K(1)
***BRAF***			
Wild type	81 (93%)		
Mutated	6 (7%)		
		Exón 15	V600E (6)
***TP53***			
Wild type	58 (66%)		
Mutated	29 (33%)		
		Exon 4	L111R (1), G112del (1)
		Exon 5	C135F (1), P152AfsTer14 (1), R156H (1), R158C (1), V173L (1), R175H (3), H178PfsTer47 (1), c.376-2A>G splice (1)
		Exon 6	Q192^*^ (1), R196^*^ (1), L201GfsTer47 (1), S215R (1).
		Exon 7	R248W (2), R248Q (1), C275Y (1), N247I (1), T253PfsTer92 (1)
		Exon 8	R282W (3), R273C (1), C277VfsTer68 (1), V272M (1)
		Exon 10	S366A (1)

Results expressed as number of cases (percentage).

### Association between the mutational profile of sCRC tumors and other features of the disease

Once sCRC patients with and without mutations in the EGFR-pathway were compared, similar disease features were observed for the two patient groups, for most parameters analyzed including therapy (Table 4). Despite this, tumors with *KRAS* mutations were more frequently localized in the right colon (p=0.05), corresponded to well-differentiated tumors (p=0.04) and displayed no lymphovascular invasion (p=0.05). In contrast *BRAF* mutated sCRC tumors were significantly associated with a poorly- or moderately- differentiated histopathology (p=0.02), peritoneal carcinomatosis (p=0.006) and microsatellite instability (p=0.007). In turn, patients who displayed *TP53* mutations showed a greater prevalence in males (p=0.05). No further statistically significant associations were found between the mutational status of *KRAS*, *NRAS*, *BRAF* and *TP53* and patient age, the histological subtype of the tumor, tumor stage, presence of lymph node involvement and metastases, tumor size, CEA serum levels and the presence of perineural invasion. For these later variables that did not achieve a statistically significant association, a post-hoc power estimation was performed which showed limited power (<0.8) to detect potential associations due to small sample size (Supplementary Table 3).

**Table 4:** Mutational status of the *KRAS*, *NRAS*, *BRAF* and *TP53* genes and their association with other clinical, biological and histopathological features of sporadic colorectal cancer (sCRC) patients (n=87)

Variable	Categories	N. of Cases (%)	*KRAS* Mutation (%)	*P-value*	*NRAS* Mutation (%)	*P-value*	*BRAF* Mutation (%)	*P-value*	*TP53* Mutation (%)	*P-value*
**Age (years)**	< 72	43 (49)	14 (33)	NS	1 (2)	NS	2 (5)	NS	18 (42)	NS
	≥ 72	44 (51)	10 (23)		0 (0)		4 (9)		11 (25)	
**Gender**	Male	51 (59)	12 (24)	NS	1 (2)	NS	3 (6)	NS	21 (41)	**0.05**
	Female	36 (41)	12 (33)		0 (0)		3 (8)		8 (22)	
**Site of primary tumor**	Right Colon	44 (51)	17 (39)		1 (2)		5 (11)		14 (32)	
	Left Colon	39 (45)	7 (18)	**0.05**	0 (0)	NS	1 (3)	NS	15 (38)	NS
	Rectum	4 (4)	0 (0)		0 (0)		0 (0)		0 (0)	
**Histological type**	Adenocarcinoma	80 (92)	24 (30)		1 (1)		5 (6)		27 (44)	
	Signet ring cell carcinoma	4 (5)	0 (0)	NS	0 (0)	NS	0 (0)	NS	1 (25)	NS
	Mucinous	2 (2)	0 (0)		0 (0)		1 (2)		1 (50)	
	adenocarcinoma									
	Neuroendocrine tumor	1 (1)	0 (0)		0 (0)		0 (0)		0 (0)	
**Grade of differentiation**	Well	24 (30)	12 (50)		0 (0)		0 (0)		11 (46)	
	Moderadate	49 (61)	10 (20)	**0.04**	1 (2)	NS	3 (6)	**0.02**	13 (27)	NS
	Poor	7 (9)	2 (29)		0 (0)		2 (29)		3 (43)	
**TNM Stage at diagnosis**	0	3 (3)	0 (0)		0 (0)		0 (0)		0 (0)	
	I	16 (19)	5 (31)		0 (0)		2 (0)		5 (31)	
	II	27 (31)	11 (41)	NS	1 (4)	NS	0 (0)	NS	8 (30)	NS
	III	35 (40)	7 (20)		0 (0)		3 (9)		13 (37)	
	IV	6 (7)	1 (17)		0 (0)		1 (17)		3 (50)	
**pN**	N0	46 (53)	16 (35)		1 (2)		2 (4)		13 (28)	
	N1	25 (29)	5 (20)	NS	0 (0)	NS	3 (12)	NS	9 (36)	NS
	N2	16 (18)	3 (19)		0 (0)		1 (16)		7 (44)	
**Metastases**	No	68 (78)	21 (31)	NS	1 (2)	NS	3 (16)	NS	23 (34)	NS
	Yes	19 (22)	3 (16)		0 (0)		3 (4)		6 (32)	
**Peritoneal carcinomatosis**	No	80 (91)	23 (29)	NS	1 (1)	NS	3 (4)	**0.006**	27 (34)	NS
	Yes	7 (9)	1 (14)		0 (0)		3 (43)		2 (29)	
**Tumor size**	<4 cm	39 (40)	9 (23)	NS	1 (2)	NS	2 (5)	NS	13 (33)	NS
	≥4 cm	48 (60)	15 (31)		0 (0)		4 (9)		16 (34)	
**CEA serum levels**	≤5 ng/ml	50 (40)	13 (26)	NS	0 (0)	NS	4 (8)	NS	19 (38)	NS
	>5 ng/ml	31 (60)	10 (32)		1 (3)		1 (3)		8 (26)	
**Lymphovascular Invasion**	No	68 (78)	22 (32)	**0.05**	1 (2)	NS	4 (6)	NS	22 (32)	
	Yes	19 (22)	2 (11)		0 (0)		2 (11)		7 (37)	NS
**Perineural Invasion**	No	65 (75)	21 (32)	NS	0 (0)	NS	4 (6)	NS	19 (29)	NS
	Yes	22 (25)	3 (14)		1 (5)		2 (9)		10 (46)	
**Microsatellite instability**	No	48 (89)	16 (33)	NS	0 (0)	NS	2 (4)	**0.007**	21 (44)	NS
	Yes	6 (11)	0 (0)		0 (0)		3 (50)		1 (17)	
**Adjuvant therapy**	No	42 (55)	16 (38)	NS	1 (2)	NS	3 (7)	NS	13 (31)	NS
	Yes	34 (45)	7 (21)		0 (0)		2 (6)		13 (38)	

^*^Results expressed as median (range) or as #number of cases (percentage); NS: statistically no significant differences detected (p >.05); CEA: carcinoembryonic antigen.

From all mutations identified only the *BRAF* mutational status showed a statistically significant association with the microsatellite status with 2/48 microsatellite stable (MSS) patients (4%) and 3/6 microsatellite instable (MSI) cases (50%) being BRAF-mutated (p=0.007).

### Prognostic impact of the *KRAS*, *NRAS*, *BRAF* and *TP53* mutational status

In total, 74/87 patients were included in survival analyses. The other 13 patients were excluded because they had sCRC tumors other than adenocarcinoma (n=7), died within the first 30 days after surgery (n=4) and/or did not undergo complete resection of the tumor (n=2). From the prognostic point of view, the *BRAF* mutation was the only mutation that had an adverse impact on PFS (median 2-year PFS of 60% *vs.* 79% for wild type *BRAF* tumors; p=0.05) (Table 5) and OS (median 2-year OS of 80% *vs.* 94% for wild type *BRAF*; p=0.001). Other clinical, biological and histopahologic characteristics of the disease that displayed a significant adverse influence on PFS in the univariate analysis included: female gender (2-year PFS rates of 63% *vs.* 90% in males; p=0.03), advanced TNM stage (2-year PFS rates of 0% for stage IV *vs.* 75% for stage III and 91% for stages 0-II ; p<0.001), and the tumor grade of differentiation (2-year PFS rates of 95% for well-differentiated *vs.* 77% for moderately- and 33% for poorly-differentiated tumors; p=0.03), the presence of lymphovascular involvement at diagnosis (2-year PFS rates of 54% *vs.* 83% for cases who showed no lymphovascular invasion; p=0.004), and perineural invasion (2-year PFS rates of 62% *vs.* 83% for cases who had no perineural invasion; p=0.03) (Table 5). Multivariate analysis of prognostic factors showed that the TNM stage at diagnosis, together with the *BRAF* mutational status were the only independent variables predicting for PFS -HR=2.77, 95%CI of 1.55-4.96 (p=0.001) and HR=4.9, 95%CI of 1.04-23.75 (p=0.05) respectively- (Table 5).

**Table 5:** Clinical, biological, genetic and therapeutic characteristics of sporadic colorectal cancer (sCRC) patients (n=74) with an impact on progression-free survival (PFS) and overall survival (OS)

Variable	PFS					OS			
	N	% 2-year PFS	Univariate analysis	Multivariate analysis	HR (95% CI)	% 2-year OS	Univariate analysis	Multivariate analysis	HR (95% CI)
**Age**									
<72 years	40	81%	NS			100%	NS		
≥72 years	34	74%				84%			
**Gender**									
Male	41	90%	0.03	NS		93%	NS		
Female	33	63%				94%			
**Site of primary tumor**									
Right colon	37	75%	NS			88%	NS		
Left colon	34	82%				97%			
Rectum	3	67%				100%			
**TNM stage at diagnosis**									
Stage 0/I/II	42	91%	<0.001	0.001	2.77(1.55-4.96)	97%	0.02	NS	
Stage III	26	75%				88%			
Stage IV	6	0%				80%			
**Grade of differentiation**									
Well	22	95%	0.03	NS		91%	NS		
Moderate	46	77%				97%			
Poor	6	33%				83%			
**CEA serum levels**									
≤5 ng/ml	45	80%	NS			96%	NS		
>5 ng/ml	24	75%				92%			
**Tumor size**									
<4 cm	44	83%	NS			100%	0.005	NS	
≥4 cm	30	70%				81%			
**Lymphovascular invasion**									
No	60	83%	0.004	NS		95%	NS		
Yes	14	54%				84%			
**Perineural invasion**									
No	57	83%	0.03	NS		93%	NS		
Yes	17	62%				93%			
**Microsatellite instability ¥**									
No	43	72%	NS			92%	0.01	NS	
Yes	5	60%				40%			
***BRAF***									
Wild type	69	79%	0.05	0.045	4.9 (1.04-23.75)	94%	0.001	0.02	4.4 (0.7-28)
Mutated	5	60%				80%			
***KRAS***									
Wild type	50	68%	NS			90%	NS		
Mutated	24	77%				100%			
***NRAS***									
Wild type	73	77%	NS			93%	NS		
Mutated	1	100%				100%			
***TP53***									
Wild type	47	81%	NS			93%	NS		
Mutated	27	77%				83%			
**Adjuvant therapy**									
No	40	84%	NS			97%	NS		
Yes	34	70%				96%			

^*^Results expressed as number of cases (percentage); NS: statistically no significant differences detected, (p >.05); CEA: carcinoembryonic antigen. ¥MSI status variable was not included in the multivariate analysis because MSI information was available only in a subset of 54 cases.

**Figure 2:**
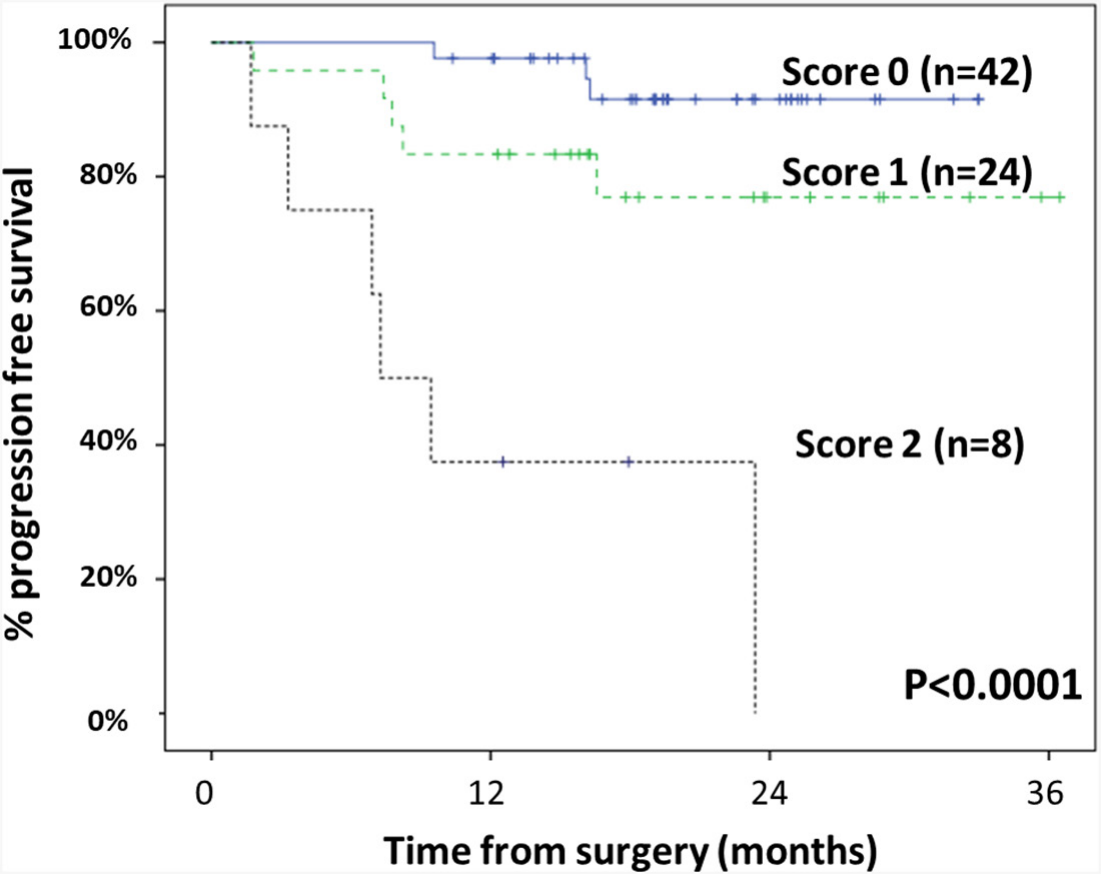
Progression-free survival (PFS) of sCRC patients stratified by the prognostic score proposed in the present study This prognostic score was established based on the most informative variables predicting for the PFS (TNM stage at diagnosis and the mutational status for the *BRAF* gene), as follows: score 0, TNM stage 0-II; score 1, TNM stage III patients with wild-type BRAF; score 2, TNM stage III cases with BRAF mutations plus TNM stage IV cases.

Based on the above results, a prognostic score was established and applied to each patient, which was based on the TNM stage and the presence vs. absence of *BRAF* mutations. Thus, patients with an early TNM stage (stage 0, I or II) were considered low-risk patients independently of their *BRAF* mutational status (score 0), whereas cases with TNM stage III and wild-type BRAF were classified into the intermediate-risk group (score 1) and patients with TNM stage III and *BRAF* mutation and those with TNM stage IV (with or without *BRAF* mutation), were considered to be high-risk (score 2). As could be expected, significantly different PFS rates were observed for cases with score 0 *vs.* score 1 *vs.* score 2: 2-year PFS of 91% *vs.* 77% *vs.* 0% respectively (p<0.0001) (Figure 2). Once the BRAF mutational status was excluded from the multivariate analyses, only TNM stage maintained statistically significance as an independent factor. Thus, no other plausible combinations were considered. Regarding OS, the TNM stage at diagnosis (p=0.02), the size of the tumor (p=0.005), and the microsatellite instability status (p=0.01) together with the *BRAF* mutational status (p=0.001), were the only individual parameters that showed an impact on patient outcome, the latter being the only variable with an independent impact on patient OS -p=0.022; HR of 4.4 (95% CI of 0.7-28)- (Table 5). However, post-hoc power estimation revealed limited power to detect additional statistically significant differences on survival analyses due to limited sample sizes (Supplementary Table 4).

### Validation of the prognostic score in an independent series of sCRC patients

In order to confirm the prognostic impact of the proposed score described above, we investigated its prognostic impact in an independent series of sCRC patients from the public GEO database (n=533). These patients were classified according to TNM stage and BRAF mutational status at diagnosis into the three previously mentioned subgroups: score 0 (n=281), score 1 (n=130) and score 2 (n=80). PFS rates were very similar for cases with score 0 *vs.* score 1 *vs.* score 2: 2-year PFS of 90% *vs.* 75% *vs.* 48% respectively (p<0.0001) (Supplementary Figure 1). These results support previous findings in our dataset.

## DISCUSSION

General consensus exists about the need to simultaneously assess multiple molecular markers for more accurate classification and personalized treatment of sCRC patients. In this study, we designed an NGS panel to investigate the frequency of *KRAS*, *NRAS*, *BRAF* and *TP53* mutations and applied it to a series of 87 sCRC patients, to determine the potential impact of the mutations detected in patient outcome, and their association with the clinical and pathological features of the disease. Overall, our results showed a good performance of the NGS panel (and method) used and the feasibility of its use in routine diagnostics. Overall, the frequency and type of the distinct mutations identified was generally concordant with previous data from the literature [17–19]. In addition, we observed that from the 4 genes investigated; only the *BRAF* V600E mutation alone or together with the TNM stage at diagnosis had an independent prognostic impact both for patient OS and PFS respectively. Although other histopathological (e.g. tumor grade, perineural and lymphovascular invasion), clinical (e.g. age) and genetic (e.g the microsatellite status) features of the disease also showed to be of potential prognostic value when individually considered, they lost their significance in the multivariate analysis.

The frequency and significance of *KRAS* mutations have been thoroughly investigated in sCRC patients, particularly in the context of anti-EGFR therapies [17, 20, 21]. Despite this, the prognostic impact of *KRAS* mutations in sCRC patients remains controversial. Thus, while some studies have found that exon 2 (codon 13) mutations predict for a worse prognosis including higher recurrence and shorter survival rates,[22] others highlighted the potential value of *KRAS* mutations in exons 3 and 4 (codons 61, 146 and 147) rather than in exon 2, as a predictive marker for PFS [23, 24] and OS [24, 25], and Palomba et al, did not find a significant prognostic impact for mutated *KRAS* in a large series of 1,284 CRC patients [26]. These apparently controversial results on the potential prognostic significance of *KRAS* mutations in sCRC, are probably due to the great genetic heterogeneity of this type of tumors, the effect of additional genetic markers and potentially also, differences in the series of patients analyzed [27]. In this regard, several studies have shown discrepancies in the *KRAS* mutational status of different tumoral samples from the same patient, and among distinct techniques. For example, Li *et al*, found a discordance rate of 19% in the frequency of *KRAS* mutations once studied by quantitative real-time PCR *vs.* MassARRAY^(®)^ techniques, between primary tumors and their paired metastatic lesions in Chinese patients with sCRC [28]. In the present study, we found KRAS mutations in around one third of the patients, particularly among well differentiated tumors, localized in the right colon, in the absence of lymphovascular invasion, in line with previous observations [29–32]. However, in our series, *KRAS* mutated sCRC patients showed similar PFS and OS rates to wild-type *KRAS* cases.

Similarly, while some previous studies have reported an association among *NRAS*-mutated patients and a shorter OS [33], we could not confirm these findings in our series, due to the fact that only 1/87 patients investigated here showed *NRAS* mutation, in line with previous studies [11]. Therefore, further studies in larger series of sCRC patients are required to establish the prognostic impact of *NRAS* mutations.

As expected, the overall frequency of *BRAF* mutations detected in our series was lower than that of *KRAS* [23, 26]. Interestingly, patients who carried *BRAF* mutations more frequently had poor-prognosis disease features such as poor-differentiated tumors [34], presence of peritoneal carcinomatosis [31, 35] and microsatellite instability, as also reported by others [32, 36]. In addition and in line with previous studies, half of the patients in our series with *BRAF* mutation were found in stage III [37]. Despite this, the *BRAF* mutation showed an independent adverse prognostic impact on both PFS and OS, as also pointed out previously by others [4, 23, 38, 39]. Further prospective studies in larger series of patients are required to confirm these observations.

*TP53* gene mutations were detected in one third of our cases, a frequency that is in the lower range of that described previously by others [40, 41]. In line with previous observations, *TP53* mutations were associated with female gender [42], but showed no impact on patient PFS and OS. In this regard, previous studies on the potential association between *TP53* mutations and the prognosis of sCRC have yielded inconsistent results [43, 44] This might be due to insufficient statistical power (in our and also other series) to detect modest survival differences between wild-type and mutated *TP53* patients, the need for longer follow-up and the potential influence of adjuvant therapy. On top of this, it should be noted that the non-mutated *TP53* allele of the gene could be functional and counteract the mutated phenotype; thus, further studies are required to investigate the potential impact of *TP5*3 genetic alterations involving the other *TP53* gene -e.g. del(17p)- [45].

As far as the slightly lower mutation frequency detected in our series, particularly KRAS, and since the level of sensitivity achieved is good, we consider it could be attributed to intratumoral heterogeneity (ITH). Tumor biopsy represents a limited fraction of the tumoral clones, simply due to spatial ITH [46].

As expected, several clinical and biological variables showed a significant association with the outcome of sCRC patients. Thus, female gender, advanced disease stage at diagnosis, poorly-differentiated tumors and the presence of lymphovascular/perineural invasion at diagnostic surgery, together with the *BRAF* mutational status, were all associated with an adverse impact on PFS in the univariate analysis, in line with previous studies [45, 47–49]. In contrast, only the TNM stage at diagnosis, the tumor size, microsatellite instability and the *BRAF* status showed a prognostic impact on OS (in the univariate analysis) among our cases. Multivariate analysis of prognostic factors showed that the best combination of independent variables for predicting PFS in sCRC patients were the presence of *BRAF* mutations and an advanced TNM stage at diagnosis, the former also retaining its independent prognostic value for OS. Based on these results, we built an original scoring system that allowed stratification of the sCRC patients analysed into three different risk groups with significantly different PFS rates at 2 years. Validation of this score in an independent dataset further strengthens this evidence. Of note, in this model, the BRAF mutational status specifically contributed to the sub-stratification of TNM stage III patients into intermediate vs. high-risk cases. Despite the fact that an association has been reported between the mutational status of genes of the *EGFR* signaling pathway and the prognosis of sCRC patients [38, 50–53], to the best of our knowledge, this is the first time that a scoring system based on combined assessment of the TNM stage at diagnosis and the *BRAF* mutational status is proposed, for the identification of sCRC patients undergoing complete tumor resection who are still at high risk of recurrence of the disease in the first 2-years after diagnosis (score 2). If the prognostic value of this new risk stratification model is confirmed in prospective series of sCRC patients it might contribute to pave the way for trials evaluating BRAF-targeted therapies in this specific subgroup of sCRC cases.

In summary, here we confirm the adverse prognosis of *BRAF* mutations in sCRC, and point out their utility, together with the TNM stage, for the identification, already at diagnosis, of a subgroup of sCRC patients (TNM stage IV plus TNM stage III and BRAF mutated patients) who, despite following complete resection of the tumor, still retain a high-risk of recurrence during the first two years after diagnosis. Additional prospective studies are required to confirm the utility of the proposed predictive model in larger series of homogeneously treated sCRC patients.

## MATERIALS AND METHODS

### Patients and samples

Freshly-frozen sCRC tissues were obtained from primary tumors of 87 Caucasian patients diagnosed with sCRC, and classified according to the World Health Organization (WHO) criteria [54], after each patient gave his/her informed consent to participate in this study. All patients underwent surgical resection of primary tumor tissues at the Department of Surgery of the University Hospital of Salamanca (HUS; Salamanca, Spain) before they had received any treatment.

Histopathological diagnosis was established by an experienced pathologist, that ensured the selection of sections representative of the tumor tissue with >70% tumor cell infiltration for further genetic analyses. DNA was extracted and isolated from freshly-frozen primary sCRC tumor tissues, using a Maxwell^®^ 16 System for Genomic DNA Extraction (Promega, Mannheim, Germany) and quantified using a Qubit dsDNA BR assay (Life Technologies, Carlsbad CA). A positive control sample -Quantitative Multiplex DNA reference standard (Horizon Discovery, Cambridge, UK)- was analyzed in parallel to each set of samples, for validation of the custom panel designed, and evaluation of the specific sensitivity of each variant call.

The study was approved by the local ethics committee of the HUS.

### Custom amplicon panel design

A custom amplicon panel for NGS analysis of the hotspot regions of *KRAS/NRAS* (exons 2, 3 and 4), and *BRAF* (exon 15), was designed; additionally, the whole *TP53* coding regions were also included in the panel for a total of 53 amplicons and 5,300bp, with an estimated coverage per sample of 7,000x.

### Preparation of DNA libraries

DNA libraries were prepared with the Truseq Custom Amplicon Panel (Illumina, San Diego, CA), according to the manufacturer´s protocol. Briefly, 50-250 ng of gDNA in 10 μl water was hybridized with a pool of oligonucleotides. Then, the unbound oligonucleotides were removed, and extension-ligation of the bound oligonucleotides was followed by PCR amplification. PCR products were cleaned and their quality checked using the 2100 Bioanalyzer (Agilent Technologies, Santa Clara, CA). A minimum size of the PCR product of ∼275 bp was required. Then, the DNA libraries were then quantified using the Qubit dsDNA HS Assay Kit and the Qubit 2.0 fluorometer (Life Technologies). Subsequently, each DNA library was diluted to a concentration of 4 nM and pooled with the other libraries in aliquots of equal volumes. The amplicon DNA libraries were paired-end sequenced using a MiSeq (Illumina) instrument.

### Analysis of DNA sequences

The sequence data generated were pre-processed with the MiSeq Reporter (MiSeq integrated software, Illumina), which uses a Burrows-Wheeler Aligner (BWA)[55] and the Genome Analysis Tool Kit (GATK) [56] for variant calling of single-nucleotide polymorphisms (SNPs) and short insertions and deletions (InDels). The identified variants were exported in the VCF data file format for further analysis using the sequencer-accompanied software (Variant Studio, Illumina) and the Integrative Genomics Viewer (IGV) software (Broad Institute, Cambridge, MA). We employed the Illumina Variant studio population frequency filters (based on the www.1000genomes.org database) to exclude variants with an overall minor allele frequency greater than 1.0%, considered common SNPs, taking as reference the Caucasian population. The following criteria were used to define and report a variant: minimum coverage of 100x, minimum variant frequency of 5%, confirmed by visual inspection using the Integrative Genomics Viewer 16 software. Detailed visual inspection with the IGV software was performed to confirm the presence and the read depth of the amplicons. Variant allele frequency (VAF) was established based on the number of reads called for the altered allele and the total number of reads called at that position by the Variant Studio Software (Illumina).

### External validation of the proposed score

A prognostic score based on the TNM stage and BRAF mutational status is established. External validation of the proposed score was performed in a previously reported group of sCRC patients from which TNM stage and BRAF mutational status at diagnosis and follow-up data were publicly available at the GEO database (accession number GSE39582) [57]. Patients included in this external validation group showed mutated BRAF in 48/533 (9%) and were classified according to the TNM staging system as follows: 38 patients had stage I (7.1%), 248 had stage II (46.5%), 186 had stage III (34.9%) and 61 had stage IV (11.4%).

### Statistical analyses

For all continuous variables, median and mean values and their standard deviation (SD) and range were calculated using the SPSS software package (SPSS 22.0 Inc, Chicago, IL); for dichotomic variables, frequencies were reported. In order to evaluate the statistical significance of differences observed between groups of mutated *vs.* non mutated patients, the Student’s T and the Mann-Whitney U tests were used for continuous variables, depending on whether they displayed or not a normal distribution, respectively. For qualitative variables, the X^2^ test was applied (cross-tab; SPSS).

For survival analyses, patients i) who had sCRC tumors other than adenocarcinoma (n=7), ii) those who dies within the first 30 days after surgery (n=4), and/or iii) did not undergo R0 resection (n=2), were excluded from the study. PFS and OS curves were plotted for the remaining 74 patients according to the method of Kaplan and Meier, and the one-sided log-rank test (one-sided) was used to establish the statistical significance of differences observed between survival curves (survival; SPSS). Multivariate analysis of prognostic factors for PFS and OS was performed using the Cox stepwise regression (forward selection) model (regression, SPSS). For multivariate analysis, only those variables showing a statistically significant association with PFS or OS in the univariate analysis, were considered. Due to the limited sample size, post-hoc power estimation was performed for all association and survival analyses and a power estimation of <0.8 was considered to be inadequate. Statistical significance was set at p values < *0.05.*

## SUPPLEMENTARY MATERIALS FIGURE AND TABLES






