# Supplementary material for: Combined assessment of the TNM stage and BRAF mutational status at diagnosis in sporadic colorectal cancer patients
Source: Oncotarget. 2018 May 8;9(35):24081–96. doi: 10.18632/oncotarget.25300 (PMC5963609; doi:10.18632/oncotarget.25300)
Supplement: Supplementary file 1 [file oncotarget-09-24081-s001.pdf]

Combined assessment of the TNM stage and *BRAF* mutational status at diagnosis in sporadic colorectal cancer patients

SUPPLEMENTARY MATERIALS

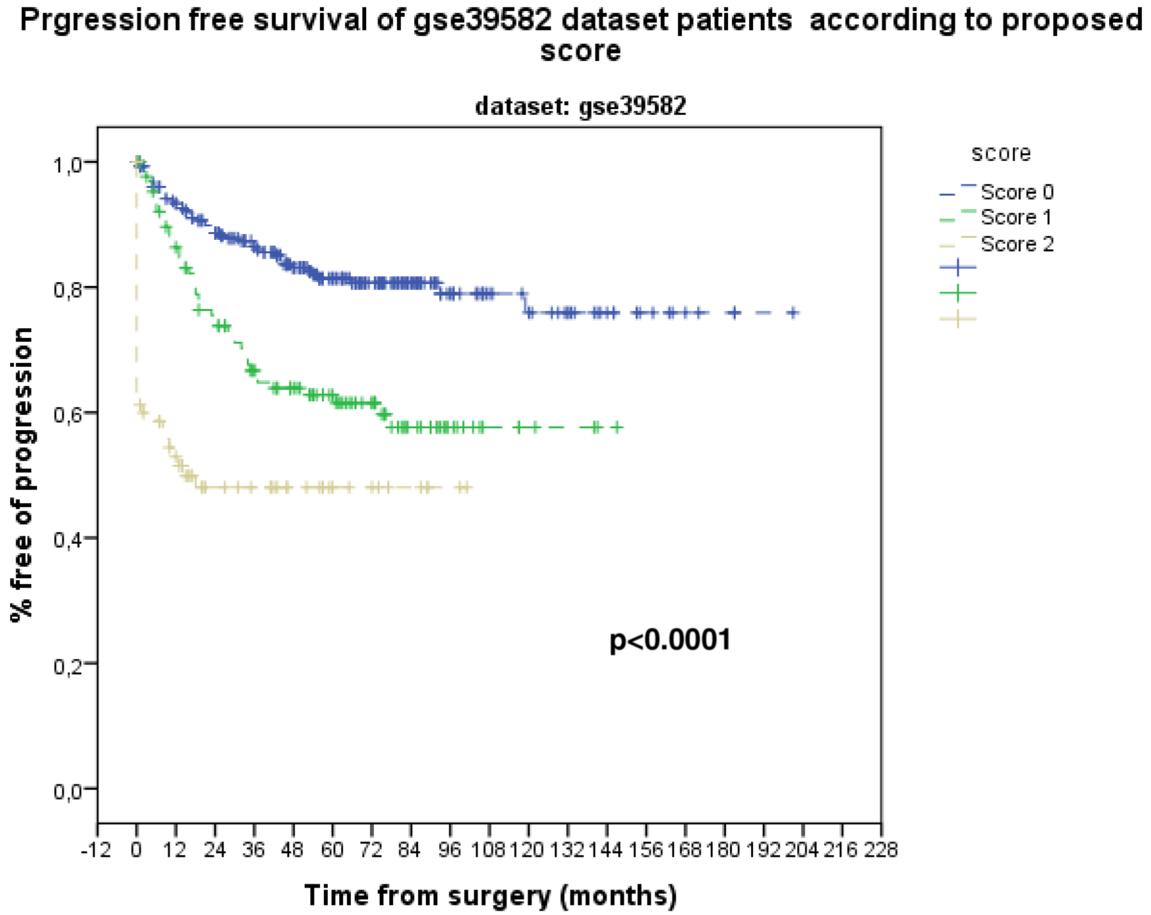

Supplementary Figure 1: Progression free survival plot of external dataset (GSE39582) of patients according to our proposed score.

Supplementary Table 1: Number of reads of each amplicon per patient

See Supplementary File 1

Supplementary Table 2: KRAS, BRAF, NRAS and TP53 variants detected and their variant allele frequency (VAF) per tumor/case analyzed, the administered treatment and microsatellite status

See Supplementary File 2

Supplementary Table 3: Ad-hoc statistical power estimation for the Chi-Square and Fisher exact tests used to estimate the significance of potential association between the distinct gene mutations identified in our patient cohort and the clinical and biological characteristics of the disease

***KRAS* mutational status**

Variable	Categories	N. of Cases (%)	KRAS mutation	P-value	Power (1-β)
Site of primary tumor	Right Colon	44 (51)	17 (39)	0.05	**1.00**
	Left Colon	39 (45)	7 (18)		
	Rectum	4 (4)	0 (0)		
Grade of differentiation	Well	24 (30)	12 (50)	0.04	**1.00**
	Moderadate	49 (61)	10 (20)		
	Poor	7 (9)	2 (29)		
Lymphovascular Invasion	No	68 (78)	22 (32)	0.05	0.43
	Yes	19 (22)	2 (11)		

***BRAF* mutational status**

Variable	Categories	N. of Cases (%)	BRAF mutation	P-value	Power (1-β)
Grade of differentiation	Well	24 (30)	0 (0)	0.02	**1.00**
	Moderadate	49 (61)	3 (6)		
	Poor	7 (9)	2 (29)		
Peritoneal carcinomatosis	No	80 (91)	3 (4)	0.006	**0.79**
	Yes	7 (9)	3 (43)		
Microsatellite instability	No	48 (89)	2 (4)	0.007	0.75
	Yes	6 (11)	3 (50)		

***TP53* mutational status**

Variable	Categories	N. of Cases (%)	TP53 mutation	P-value	Power (1-β)
Gender	Male	51 (59)	21 (41)	0.05	0.54
	Female	36 (41)	8 (22)		

Bold letters indicate adequate statistical power (Power (1-β)> 0.8).
Only variables that displayed a statistical significant association with the mutational status of the different genes investigated are included in this table. Other clinical and biological disease features statistically not significantly associated with the mutational status for the investigated genes showed a statistical power below the established threshold of >0.80, due to small sample size.

Supplementary Table 4: Statistical power calculation for progression free survival (PFS) and overall survival (OS) based on the log-rank test

**Progression free survival**				
**Variable**	**N. of cases**	**% 2-year PFS**	***P*-value**	**Power (1-β) (2-tail)**
Gender				
Male	41	90%	0.03	**0.77**
Female	33	63%		
TNM stage at diagnosis				
Stage 0/I/II	42	91%	<0.001	0.43
Stage III	26	75%		NAN
Stage IV	6	0%		NAN
Grade of differentiation				
Well	22	95%	0.03	0.41
Moderate	46	77%		**0.94**
Poor	6	33%		**0.82**
Lymphovascular invasion				
No	60	83%	0.004	0.73
Yes	14	54%		
Perineural invasion				
No	57	83%	0.03	0.49
Yes	17	62%		
BRAF				
Wild type	69	79%	0.05	0.20
Mutated	5	60%		
**Overall survival**				
**Variable**	**N. of cases**	**% 2-year OS**	***P*-value**	**Power (1-β) (2-tail)**
TNM stage at diagnosis				
Stage 0/I/II	42	97%	0.02	0.31
Stage III	26	88%		0.45
Stage IV	6	80%		0.08
Tumor size				
<4 cm	44	100%	0.005	0.00
≥4 cm	30	81%		
Microsatellite instability				
No	43	92%	0.01	**0.97**
Yes	5	40%		
BRAF				
Wild type	69	94%	0.001	0.24
Mutated	5	80%		

NAN: it is not possible to calculate this value.
Bold letter indicate adequate statistical power (Power (1-β)> 0.8).
