# Supplementary material for: Combined assessment of the TNM stage and BRAF mutational status at diagnosis in sporadic colorectal cancer patients
Source: Oncotarget. 2018 May 8;9(35):24081–96. doi: 10.18632/oncotarget.25300 (PMC5963609; doi:10.18632/oncotarget.25300)
Supplement: Supplementary file 2 [file oncotarget-09-24081-s002.docx]

**Supplementary Table 1:** Number of reads of each amplicon per patient.

| **amplicon** | **GENE** | **REF SAMPLE** | **P1** | **P2** | **P3** | **P4** | **P5** |
| --- | --- | --- | --- | --- | --- | --- | --- |
| 1 | BRAF1 | 2191 | 1426 | 1493 | 2923 | 1999 | 1791 |
| 2 | BRAF2 | 6650 | 6430 | 5251 | 9508 | 7817 | 6559 |
| 3 | NRAS1 | 2401 | 3376 | 2965 | 4866 | 3525 | 3589 |
| 4 | NRAS2 | 2331 | 3346 | 2652 | 5153 | 3450 | 3802 |
| 5 | NRAS3 | 2232 | 3808 | 3508 | 5225 | 4095 | 3950 |
| 6 | NRAS4 | 860 | 2377 | 2 | 2167 | 453 | 402 |
| 7 | NRAS5 | 2048 | 3282 | 2640 | 4834 | 3563 | 3566 |
| 8 | KRAS1 | 1934 | 4969 | 3556 | 4650 | 4058 | 3575 |
| 9 | KRAS2 | 1170 | 2186 | 1424 | 2102 | 1627 | 1530 |
| 10 | KRAS3 | 1141 | 3207 | 2257 | 3727 | 2988 | 2019 |
| 11 | KRAS4 | 1889 | 3958 | 3270 | 5175 | 4246 | 3856 |
| 12 | KRAS5 | 1815 | 3420 | 2893 | 3183 | 2866 | 2520 |
| 13 | KRAS6 | 1805 | 3725 | 3108 | 4468 | 3688 | 3582 |
| 14 | KRAS7 | 1591 | 4454 | 3761 | 4869 | 4026 | 3442 |
| 15 | TP53_1 | 4356 | 5571 | 4591 | 5867 | 7534 | 8304 |
| 16 | TP53_2 | 1431 | 2354 | 2352 | 1873 | 2686 | 2500 |
| 17 | TP53_3 | 579 | 574 | 933 | 331 | 1013 | 647 |
| 18 | TP53_4 | 694 | 1770 | 1930 | 1192 | 1724 | 1798 |
| 19 | TP53_5 | 1516 | 1868 | 1616 | 1018 | 1236 | 1292 |
| 20 | TP53_6 | 1256 | 1627 | 1707 | 1508 | 1810 | 1561 |
| 21 | TP53_7 | 1483 | 2679 | 2670 | 1893 | 2078 | 2024 |
| 22 | TP53_8 | 134 | 105 | 164 | 107 | 94 | 85 |
| 23 | TP53_9 | 2848 | 2777 | 2572 | 2630 | 3152 | 3330 |
| 24 | TP53_10 | 1054 | 1857 | 1482 | 2432 | 2850 | 3264 |
| 25 | TP53_11 | 3022 | 6132 | 5571 | 5400 | 6586 | 6713 |
| 26 | TP53_12 | 270 | 354 | 397 | 219 | 335 | 431 |
| 27 | TP53_13 | 3964 | 3904 | 5045 | 3550 | 4501 | 4604 |
| 28 | TP53_14 | 8568 | 9160 | 8284 | 8853 | 11097 | 12280 |
| 29 | TP53_15 | 4950 | 6355 | 5776 | 5046 | 6404 | 6452 |
| 30 | TP53_16 | 207 | 166 | 263 | 107 | 131 | 146 |
| 31 | TP53_17 | 4482 | 4242 | 4231 | 4486 | 5878 | 6107 |
| 32 | TP53_18 | 1989 | 2803 | 2531 | 1870 | 2089 | 2 |
| 33 | TP53_19 | 3901 | 5311 | 4864 | 5696 | 7444 | 8569 |
| 34 | TP53_20 | 3269 | 5020 | 4891 | 4525 | 6351 | 7514 |
| 35 | TP53_21 | 2087 | 3090 | 3356 | 2651 | 3378 | 3964 |
| 36 | TP53_22 | 65 | 88 | 144 | 58 | 82 | 87 |
| 37 | TP53_23 | 3592 | 5438 | 5123 | 4707 | 6530 | 7036 |
| 38 | TP53_24 | 2118 | 3603 | 3019 | 3568 | 4829 | 5341 |
| 39 | TP53_25 | 2880 | 3757 | 3334 | 2967 | 4067 | 3822 |
| 40 | TP53_26 | 763 | 1062 | 1183 | 633 | 916 | 976 |
| 41 | TP53_27 | 317 | 299 | 234 | 145 | 392 | 197 |
| 42 | TP53_28 | 477 | 1155 | 1210 | 926 | 1427 | 1204 |
| 43 | TP53_29 | 64 | 113 | 216 | 75 | 78 | 119 |
| 44 | TP53_30 | 95 | 162 | 224 | 114 | 150 | 180 |
| 45 | TP53_31 | 1284 | 1479 | 1578 | 1746 | 2143 | 2452 |
| 46 | TP53_32 | 4858 | 5987 | 4664 | 4972 | 6202 | 6790 |
| 47 | TP53_33 | 1593 | 2139 | 2190 | 1524 | 1811 | 2068 |
| 48 | TP53_34 | 2384 | 3631 | 3467 | 3904 | 4664 | 5093 |
| 49 | TP53_35 | 1793 | 2121 | 2047 | 2578 | 3674 | 4092 |
| 50 | TP53_36 | 3653 | 4711 | 4881 | 4266 | 5611 | 6335 |
| 51 | TP53_37 | 6043 | 8124 | 6720 | 6581 | 7833 | 8661 |
| 52 | TP53_38 | 41 | 114 | 71 | 136 | 175 | 178 |
| 53 | TP53_39 | 3869 | 4057 | 3478 | 3952 | 5135 | 5779 |

| **amplicon** | **GENE** | **P6** | **P7** | **P8** | **P9** | **P10** | **P11** |
| --- | --- | --- | --- | --- | --- | --- | --- |
| 1 | BRAF1 | 2239 | 2091 | 1253 | 2186 | 1852 | 1941 |
| 2 | BRAF2 | 7467 | 8872 | 6999 | 7031 | 5745 | 7452 |
| 3 | NRAS1 | 3818 | 3771 | 3905 | 3380 | 3483 | 3243 |
| 4 | NRAS2 | 4115 | 3714 | 3875 | 3800 | 4049 | 3819 |
| 5 | NRAS3 | 4731 | 4235 | 4661 | 2774 | 3405 | 3064 |
| 6 | NRAS4 | 2187 | 13 | 734 | 2183 | 417 | 479 |
| 7 | NRAS5 | 3745 | 3311 | 2727 | 3055 | 3450 | 2965 |
| 8 | KRAS1 | 5036 | 4515 | 2694 | 2465 | 2742 | 3114 |
| 9 | KRAS2 | 2087 | 1691 | 836 | 1061 | 1381 | 1595 |
| 10 | KRAS3 | 3788 | 1929 | 2038 | 1649 | 2093 | 1535 |
| 11 | KRAS4 | 5278 | 4742 | 4018 | 2859 | 3053 | 2919 |
| 12 | KRAS5 | 3612 | 3426 | 2253 | 1971 | 2130 | 2457 |
| 13 | KRAS6 | 4739 | 3804 | 3029 | 2228 | 1369 | 2518 |
| 14 | KRAS7 | 5144 | 4334 | 3193 | 2259 | 2390 | 2910 |
| 15 | TP53_1 | 5949 | 7228 | 8883 | 5890 | 6690 | 5092 |
| 16 | TP53_2 | 2349 | 2247 | 2344 | 1415 | 1759 | 1713 |
| 17 | TP53_3 | 435 | 470 | 1207 | 343 | 416 | 454 |
| 18 | TP53_4 | 1533 | 1753 | 2085 | 1172 | 1395 | 1207 |
| 19 | TP53_5 | 993 | 1193 | 924 | 770 | 1219 | 1130 |
| 20 | TP53_6 | 1255 | 1764 | 2015 | 1461 | 1476 | 1434 |
| 21 | TP53_7 | 1765 | 2350 | 2198 | 1421 | 1705 | 1702 |
| 22 | TP53_8 | 54 | 156 | 175 | 120 | 86 | 75 |
| 23 | TP53_9 | 2591 | 2843 | 2079 | 2130 | 2593 | 2339 |
| 24 | TP53_10 | 2155 | 2585 | 2207 | 2077 | 2601 | 1701 |
| 25 | TP53_11 | 5472 | 5917 | 6200 | 4189 | 5368 | 4517 |
| 26 | TP53_12 | 285 | 328 | 459 | 228 | 320 | 236 |
| 27 | TP53_13 | 3502 | 4027 | 5343 | 4155 | 4145 | 3085 |
| 28 | TP53_14 | 8272 | 10582 | 13473 | 8121 | 9620 | 7845 |
| 29 | TP53_15 | 5152 | 6412 | 6792 | 4703 | 5458 | 4526 |
| 30 | TP53_16 | 106 | 144 | 177 | 97 | 156 | 104 |
| 31 | TP53_17 | 4382 | 5492 | 7168 | 4557 | 5329 | 4000 |
| 32 | TP53_18 | 1965 | 1877 | 2679 | 1777 | 1054 | 1458 |
| 33 | TP53_19 | 6030 | 7323 | 9080 | 4624 | 5950 | 4828 |
| 34 | TP53_20 | 5003 | 5672 | 8147 | 3049 | 4602 | 3645 |
| 35 | TP53_21 | 2659 | 3621 | 4461 | 2457 | 3061 | 2609 |
| 36 | TP53_22 | 54 | 49 | 135 | 72 | 62 | 62 |
| 37 | TP53_23 | 5170 | 6335 | 6942 | 4021 | 4746 | 3902 |
| 38 | TP53_24 | 3791 | 4320 | 5433 | 2253 | 3105 | 2619 |
| 39 | TP53_25 | 3008 | 3803 | 4473 | 2759 | 3059 | 2910 |
| 40 | TP53_26 | 694 | 814 | 766 | 454 | 703 | 732 |
| 41 | TP53_27 | 272 | 360 | 378 | 283 | 137 | 133 |
| 42 | TP53_28 | 947 | 1337 | 1368 | 733 | 790 | 886 |
| 43 | TP53_29 | 50 | 88 | 98 | 95 | 118 | 89 |
| 44 | TP53_30 | 76 | 157 | 255 | 105 | 136 | 101 |
| 45 | TP53_31 | 1685 | 1780 | 2440 | 1303 | 1586 | 1295 |
| 46 | TP53_32 | 5059 | 5629 | 5690 | 4104 | 5148 | 4423 |
| 47 | TP53_33 | 1479 | 1813 | 2082 | 1543 | 1856 | 1800 |
| 48 | TP53_34 | 3717 | 4436 | 5257 | 2557 | 3457 | 2851 |
| 49 | TP53_35 | 2808 | 3485 | 4290 | 2066 | 2657 | 1927 |
| 50 | TP53_36 | 4744 | 5326 | 6921 | 3715 | 4413 | 3810 |
| 51 | TP53_37 | 6489 | 7210 | 6979 | 5054 | 6754 | 6068 |
| 52 | TP53_38 | 160 | 137 | 197 | 118 | 107 | 85 |
| 53 | TP53_39 | 4195 | 4377 | 6608 | 4033 | 4479 | 4532 |

| **amplicon** | **GENE** | **P12** | **P13** | **P14** | **P15** | **P16** | **P17** |
| --- | --- | --- | --- | --- | --- | --- | --- |
| 1 | BRAF1 | 2065 | 2039 | 1641 | 2278 | 3088 | 2461 |
| 2 | BRAF2 | 8094 | 7670 | 5560 | 8090 | 7845 | 9133 |
| 3 | NRAS1 | 3850 | 3969 | 3202 | 3195 | 3766 | 7565 |
| 4 | NRAS2 | 3719 | 3999 | 2996 | 3662 | 4503 | 4625 |
| 5 | NRAS3 | 3570 | 4163 | 3175 | 2971 | 3874 | 4470 |
| 6 | NRAS4 | 1656 | 1698 | 531 | 1153 | 2726 | 1342 |
| 7 | NRAS5 | 3163 | 3652 | 3050 | 3132 | 4357 | 4694 |
| 8 | KRAS1 | 2472 | 4401 | 2692 | 3066 | 4692 | 4832 |
| 9 | KRAS2 | 1086 | 2210 | 1484 | 1608 | 3648 | 2188 |
| 10 | KRAS3 | 1726 | 1703 | 2208 | 1435 | 3123 | 2809 |
| 11 | KRAS4 | 3223 | 4478 | 3225 | 3899 | 3546 | 5258 |
| 12 | KRAS5 | 1988 | 3044 | 2631 | 2338 | 2990 | 4364 |
| 13 | KRAS6 | 3088 | 4056 | 3324 | 3556 | 4409 | 5571 |
| 14 | KRAS7 | 2261 | 3227 | 2670 | 2882 | 3195 | 5188 |
| 15 | TP53_1 | 6257 | 7733 | 6652 | 7000 | 5408 | 11765 |
| 16 | TP53_2 | 1674 | 2073 | 1898 | 1827 | 2260 | 3846 |
| 17 | TP53_3 | 439 | 470 | 463 | 1119 | 832 | 995 |
| 18 | TP53_4 | 1033 | 1572 | 1190 | 1427 | 2099 | 2676 |
| 19 | TP53_5 | 867 | 1368 | 1186 | 1077 | 1791 | 2937 |
| 20 | TP53_6 | 1397 | 1873 | 1334 | 1236 | 1824 | 2863 |
| 21 | TP53_7 | 1303 | 1800 | 1791 | 1479 | 2856 | 3649 |
| 22 | TP53_8 | 100 | 94 | 126 | 100 | 162 | 227 |
| 23 | TP53_9 | 2136 | 3537 | 2810 | 2968 | 4018 | 6093 |
| 24 | TP53_10 | 2286 | 2762 | 2378 | 2476 | 3030 | 4765 |
| 25 | TP53_11 | 3502 | 5117 | 4149 | 4966 | 6058 | 11524 |
| 26 | TP53_12 | 230 | 336 | 226 | 326 | 394 | 589 |
| 27 | TP53_13 | 3369 | 4277 | 4305 | 4582 | 6019 | 10125 |
| 28 | TP53_14 | 7763 | 9841 | 8650 | 8977 | 10611 | 18942 |
| 29 | TP53_15 | 4565 | 6515 | 5512 | 5228 | 7475 | 11852 |
| 30 | TP53_16 | 81 | 132 | 145 | 131 | 423 | 319 |
| 31 | TP53_17 | 5044 | 6059 | 4837 | 5251 | 6012 | 11153 |
| 32 | TP53_18 | 1337 | 2595 | 1972 | 2197 | 2816 | 5710 |
| 33 | TP53_19 | 5552 | 6293 | 5604 | 5382 | 5034 | 11534 |
| 34 | TP53_20 | 4373 | 4812 | 3945 | 4154 | 4057 | 9837 |
| 35 | TP53_21 | 2366 | 3446 | 2711 | 3268 | 3913 | 6438 |
| 36 | TP53_22 | 66 | 66 | 77 | 87 | 98 | 202 |
| 37 | TP53_23 | 4109 | 4903 | 3611 | 4396 | 5552 | 6781 |
| 38 | TP53_24 | 2984 | 3537 | 2457 | 2856 | 2720 | 4360 |
| 39 | TP53_25 | 2535 | 3294 | 2979 | 2884 | 3787 | 6851 |
| 40 | TP53_26 | 515 | 841 | 674 | 602 | 1615 | 1800 |
| 41 | TP53_27 | 295 | 188 | 199 | 229 | 444 | 443 |
| 42 | TP53_28 | 887 | 1052 | 912 | 878 | 1519 | 1933 |
| 43 | TP53_29 | 65 | 163 | 157 | 105 | 121 | 270 |
| 44 | TP53_30 | 81 | 141 | 186 | 120 | 110 | 503 |
| 45 | TP53_31 | 1526 | 1890 | 1359 | 1425 | 2103 | 3082 |
| 46 | TP53_32 | 4464 | 5935 | 4976 | 4716 | 7823 | 11774 |
| 47 | TP53_33 | 1205 | 1864 | 1408 | 1687 | 3639 | 3995 |
| 48 | TP53_34 | 3046 | 3797 | 3181 | 3230 | 3667 | 7022 |
| 49 | TP53_35 | 2668 | 2934 | 2218 | 2191 | 2732 | 4602 |
| 50 | TP53_36 | 3747 | 4822 | 4023 | 3958 | 4752 | 9434 |
| 51 | TP53_37 | 5407 | 7723 | 6753 | 6401 | 8970 | 14755 |
| 52 | TP53_38 | 138 | 135 | 135 | 125 | 224 | 196 |
| 53 | TP53_39 | 3770 | 4331 | 3890 | 4254 | 3283 | 7678 |

| **amplicon** | **GENE** | **P18** | **P19** | **P20** | **P21** | **P22** | **P23** |
| --- | --- | --- | --- | --- | --- | --- | --- |
| 1 | BRAF1 | 1807 | 2443 | 2119 | 1985 | 2086 | 5253 |
| 2 | BRAF2 | 5673 | 6737 | 8384 | 7627 | 6209 | 17165 |
| 3 | NRAS1 | 3722 | 3755 | 4733 | 4389 | 3763 | 5956 |
| 4 | NRAS2 | 3788 | 3832 | 4558 | 4577 | 4268 | 5620 |
| 5 | NRAS3 | 3346 | 3269 | 4229 | 4507 | 3543 | 5039 |
| 6 | NRAS4 | 513 | 581 | 701 | 1984 | 1779 | 463 |
| 7 | NRAS5 | 3450 | 3266 | 4208 | 4296 | 3533 | 5219 |
| 8 | KRAS1 | 2565 | 3021 | 3978 | 4193 | 3619 | 5490 |
| 9 | KRAS2 | 1388 | 1598 | 1992 | 2088 | 1874 | 3299 |
| 10 | KRAS3 | 1653 | 1327 | 2474 | 3003 | 2513 | 3969 |
| 11 | KRAS4 | 2657 | 3387 | 3962 | 3966 | 3288 | 5734 |
| 12 | KRAS5 | 2637 | 2116 | 2938 | 3085 | 2643 | 4427 |
| 13 | KRAS6 | 3351 | 2962 | 3514 | 3983 | 3289 | 5779 |
| 14 | KRAS7 | 3013 | 2844 | 3526 | 3611 | 2795 | 5119 |
| 15 | TP53_1 | 5489 | 5363 | 7115 | 7293 | 6428 | 8110 |
| 16 | TP53_2 | 2083 | 1930 | 2450 | 2418 | 2205 | 3006 |
| 17 | TP53_3 | 574 | 427 | 568 | 519 | 575 | 741 |
| 18 | TP53_4 | 1537 | 1258 | 1673 | 1673 | 1656 | 2452 |
| 19 | TP53_5 | 1683 | 1127 | 1484 | 1737 | 1432 | 1944 |
| 20 | TP53_6 | 1360 | 1609 | 1893 | 1846 | 1820 | 1876 |
| 21 | TP53_7 | 1906 | 1596 | 2301 | 2095 | 2098 | 2603 |
| 22 | TP53_8 | 100 | 116 | 134 | 139 | 123 | 115 |
| 23 | TP53_9 | 3662 | 2786 | 3801 | 4073 | 3266 | 4683 |
| 24 | TP53_10 | 2757 | 2262 | 2972 | 3302 | 2732 | 4017 |
| 25 | TP53_11 | 5859 | 5116 | 6560 | 7263 | 6178 | 8079 |
| 26 | TP53_12 | 360 | 265 | 280 | 392 | 293 | 433 |
| 27 | TP53_13 | 5648 | 4147 | 5351 | 5338 | 5378 | 7113 |
| 28 | TP53_14 | 10069 | 7692 | 10481 | 11470 | 9396 | 12957 |
| 29 | TP53_15 | 6149 | 5240 | 7441 | 7420 | 7061 | 9176 |
| 30 | TP53_16 | 177 | 104 | 166 | 181 | 153 | 258 |
| 31 | TP53_17 | 5732 | 4378 | 6329 | 6520 | 5480 | 7128 |
| 32 | TP53_18 | 2566 | 2191 | 2633 | 3023 | 2761 | 3374 |
| 33 | TP53_19 | 5967 | 4926 | 6498 | 7198 | 6050 | 7393 |
| 34 | TP53_20 | 4860 | 3817 | 5342 | 5767 | 4727 | 6244 |
| 35 | TP53_21 | 3298 | 2836 | 3572 | 3834 | 3616 | 4821 |
| 36 | TP53_22 | 66 | 72 | 87 | 102 | 96 | 136 |
| 37 | TP53_23 | 3908 | 4368 | 5601 | 5907 | 5178 | 6676 |
| 38 | TP53_24 | 2436 | 2734 | 3423 | 3897 | 2741 | 3690 |
| 39 | TP53_25 | 3454 | 2735 | 3856 | 3717 | 3286 | 4541 |
| 40 | TP53_26 | 1079 | 768 | 1021 | 1436 | 982 | 1323 |
| 41 | TP53_27 | 174 | 206 | 174 | 361 | 290 | 421 |
| 42 | TP53_28 | 1204 | 854 | 993 | 1311 | 1041 | 1360 |
| 43 | TP53_29 | 177 | 77 | 110 | 106 | 99 | 139 |
| 44 | TP53_30 | 230 | 105 | 137 | 224 | 129 | 166 |
| 45 | TP53_31 | 1576 | 1389 | 1829 | 2001 | 1771 | 2381 |
| 46 | TP53_32 | 5962 | 4965 | 6779 | 7432 | 6758 | 8573 |
| 47 | TP53_33 | 2178 | 1997 | 2275 | 2245 | 2345 | 3055 |
| 48 | TP53_34 | 3633 | 3261 | 4352 | 4791 | 4154 | 5208 |
| 49 | TP53_35 | 2493 | 2223 | 3089 | 3372 | 2703 | 3391 |
| 50 | TP53_36 | 4713 | 4123 | 5541 | 5970 | 5445 | 6882 |
| 51 | TP53_37 | 7981 | 6584 | 8864 | 9235 | 8456 | 10566 |
| 52 | TP53_38 | 123 | 116 | 137 | 153 | 121 | 173 |
| 53 | TP53_39 | 3891 | 3328 | 4645 | 4880 | 4143 | 5139 |

| **amplicon** | **GENE** | **P24** | **P25** | **P26** | **P27** | **P28** | **P29** |
| --- | --- | --- | --- | --- | --- | --- | --- |
| 1 | BRAF1 | 1590 | 2439 | 1868 | 2609 | 3195 | 2713 |
| 2 | BRAF2 | 8159 | 8630 | 7686 | 9342 | 13987 | 9792 |
| 3 | NRAS1 | 3855 | 4301 | 5169 | 5341 | 6135 | 4071 |
| 4 | NRAS2 | 3997 | 4342 | 5095 | 4025 | 6061 | 5139 |
| 5 | NRAS3 | 3460 | 3885 | 3923 | 3664 | 5695 | 4343 |
| 6 | NRAS4 | 1615 | 3663 | 218 | 198 | 2912 | 2445 |
| 7 | NRAS5 | 2917 | 3422 | 3634 | 2927 | 4921 | 3673 |
| 8 | KRAS1 | 4047 | 3424 | 4160 | 3331 | 5167 | 4453 |
| 9 | KRAS2 | 1738 | 1697 | 2289 | 1573 | 2416 | 2330 |
| 10 | KRAS3 | 2979 | 2379 | 3124 | 2162 | 2503 | 3382 |
| 11 | KRAS4 | 3716 | 2810 | 3839 | 3120 | 4492 | 3934 |
| 12 | KRAS5 | 3290 | 2508 | 2967 | 2965 | 4215 | 3239 |
| 13 | KRAS6 | 3295 | 3332 | 4236 | 3905 | 4738 | 4510 |
| 14 | KRAS7 | 3381 | 2837 | 3392 | 3098 | 3885 | 3441 |
| 15 | TP53_1 | 6980 | 6692 | 8357 | 7786 | 11938 | 8133 |
| 16 | TP53_2 | 1926 | 2299 | 3023 | 2632 | 3342 | 2787 |
| 17 | TP53_3 | 703 | 1500 | 1675 | 886 | 984 | 2769 |
| 18 | TP53_4 | 1849 | 2334 | 2262 | 2191 | 2765 | 2411 |
| 19 | TP53_5 | 1125 | 1470 | 1496 | 1728 | 1993 | 1693 |
| 20 | TP53_6 | 1658 | 1778 | 1706 | 1795 | 3076 | 1651 |
| 21 | TP53_7 | 2852 | 2032 | 2502 | 2825 | 4242 | 2407 |
| 22 | TP53_8 | 164 | 117 | 101 | 198 | 297 | 131 |
| 23 | TP53_9 | 2144 | 3260 | 3701 | 3406 | 4349 | 3476 |
| 24 | TP53_10 | 2202 | 3040 | 2975 | 2819 | 4460 | 2921 |
| 25 | TP53_11 | 4371 | 6065 | 6822 | 6094 | 8696 | 6179 |
| 26 | TP53_12 | 300 | 455 | 435 | 359 | 608 | 419 |
| 27 | TP53_13 | 4265 | 5743 | 5596 | 5597 | 6796 | 5767 |
| 28 | TP53_14 | 10876 | 12790 | 14239 | 11819 | 17915 | 12874 |
| 29 | TP53_15 | 7545 | 9270 | 9627 | 7551 | 12758 | 9318 |
| 30 | TP53_16 | 198 | 160 | 262 | 174 | 302 | 274 |
| 31 | TP53_17 | 5917 | 7079 | 7404 | 6991 | 9755 | 7904 |
| 32 | TP53_18 | 2553 | 2609 | 3091 | 2360 | 3872 | 3440 |
| 33 | TP53_19 | 6987 | 7859 | 9687 | 7927 | 12054 | 8407 |
| 34 | TP53_20 | 4986 | 5996 | 6033 | 6087 | 8688 | 5701 |
| 35 | TP53_21 | 3904 | 4029 | 5009 | 4253 | 6637 | 4773 |
| 36 | TP53_22 | 95 | 172 | 181 | 73 | 144 | 155 |
| 37 | TP53_23 | 5891 | 6970 | 7725 | 6233 | 9301 | 7110 |
| 38 | TP53_24 | 2552 | 3503 | 3716 | 3447 | 4897 | 3784 |
| 39 | TP53_25 | 2807 | 3113 | 3749 | 3623 | 5546 | 3639 |
| 40 | TP53_26 | 1216 | 1630 | 1656 | 1542 | 2138 | 1831 |
| 41 | TP53_27 | 249 | 412 | 256 | 431 | 696 | 462 |
| 42 | TP53_28 | 933 | 1474 | 1606 | 1406 | 2025 | 1492 |
| 43 | TP53_29 | 235 | 233 | 258 | 183 | 237 | 212 |
| 44 | TP53_30 | 198 | 100 | 121 | 103 | 177 | 111 |
| 45 | TP53_31 | 1624 | 1870 | 2023 | 1903 | 2617 | 2112 |
| 46 | TP53_32 | 6445 | 7638 | 9268 | 8072 | 11021 | 8885 |
| 47 | TP53_33 | 1665 | 2602 | 2701 | 2517 | 3262 | 2785 |
| 48 | TP53_34 | 2704 | 3522 | 4334 | 3625 | 5271 | 3639 |
| 49 | TP53_35 | 3399 | 3295 | 4076 | 3465 | 5390 | 3601 |
| 50 | TP53_36 | 5555 | 6746 | 7381 | 6515 | 10483 | 7187 |
| 51 | TP53_37 | 7632 | 9817 | 10693 | 9369 | 13596 | 10225 |
| 52 | TP53_38 | 191 | 189 | 184 | 165 | 290 | 240 |
| 53 | TP53_39 | 4017 | 4573 | 4833 | 4603 | 6709 | 4500 |

| **amplicon** | **GENE** | **P30** | **P31** | **P32** | **P33** | **P34** | **P35** |
| --- | --- | --- | --- | --- | --- | --- | --- |
| 1 | BRAF1 | 3576 | 2702 | 3608 | 2223 | 2341 | 2378 |
| 2 | BRAF2 | 13015 | 10588 | 11062 | 6591 | 8804 | 7510 |
| 3 | NRAS1 | 5992 | 2840 | 4756 | 3035 | 5033 | 5191 |
| 4 | NRAS2 | 5758 | 3267 | 5589 | 3857 | 4777 | 5063 |
| 5 | NRAS3 | 5360 | 2302 | 4345 | 2525 | 4409 | 4653 |
| 6 | NRAS4 | 221 | 2134 | 106 | 2127 | 2358 | 2076 |
| 7 | NRAS5 | 4529 | 3142 | 3719 | 2255 | 3664 | 3634 |
| 8 | KRAS1 | 4902 | 2303 | 3608 | 2822 | 4394 | 4192 |
| 9 | KRAS2 | 2519 | 1308 | 1877 | 1522 | 2377 | 2015 |
| 10 | KRAS3 | 3522 | 1825 | 2405 | 1558 | 1946 | 2968 |
| 11 | KRAS4 | 3744 | 2987 | 2963 | 2788 | 3727 | 3693 |
| 12 | KRAS5 | 3247 | 1541 | 2866 | 2367 | 3559 | 2689 |
| 13 | KRAS6 | 4616 | 3793 | 4064 | 3024 | 4056 | 4210 |
| 14 | KRAS7 | 3692 | 1474 | 2943 | 2602 | 3134 | 3184 |
| 15 | TP53_1 | 7926 | 6445 | 5306 | 5922 | 7372 | 9122 |
| 16 | TP53_2 | 2992 | 1827 | 1500 | 1974 | 2247 | 2427 |
| 17 | TP53_3 | 1439 | 348 | 500 | 702 | 779 | 1938 |
| 18 | TP53_4 | 2471 | 910 | 1158 | 1244 | 1850 | 2422 |
| 19 | TP53_5 | 1477 | 478 | 862 | 1109 | 1664 | 1812 |
| 20 | TP53_6 | 1887 | 1258 | 1243 | 1232 | 1911 | 1729 |
| 21 | TP53_7 | 2757 | 1233 | 1713 | 2118 | 2518 | 2259 |
| 22 | TP53_8 | 206 | 115 | 151 | 106 | 129 | 77 |
| 23 | TP53_9 | 3782 | 2107 | 2072 | 2462 | 3196 | 3812 |
| 24 | TP53_10 | 3077 | 2410 | 1907 | 1957 | 2532 | 3211 |
| 25 | TP53_11 | 6774 | 3759 | 3538 | 3816 | 5102 | 6224 |
| 26 | TP53_12 | 523 | 248 | 198 | 202 | 256 | 356 |
| 27 | TP53_13 | 6012 | 4160 | 3743 | 3863 | 4791 | 6229 |
| 28 | TP53_14 | 13093 | 9572 | 8073 | 10785 | 11727 | 14383 |
| 29 | TP53_15 | 9368 | 4849 | 5442 | 5954 | 8320 | 9960 |
| 30 | TP53_16 | 247 | 105 | 174 | 136 | 269 | 291 |
| 31 | TP53_17 | 6757 | 4939 | 5284 | 5360 | 7046 | 8532 |
| 32 | TP53_18 | 3217 | 1198 | 1778 | 2177 | 2927 | 1950 |
| 33 | TP53_19 | 8574 | 5980 | 4847 | 5750 | 7117 | 8780 |
| 34 | TP53_20 | 6038 | 5165 | 3519 | 3574 | 5152 | 5884 |
| 35 | TP53_21 | 4707 | 2783 | 2483 | 2911 | 3982 | 4774 |
| 36 | TP53_22 | 115 | 89 | 112 | 72 | 128 | 148 |
| 37 | TP53_23 | 7344 | 4865 | 4187 | 4404 | 5319 | 6930 |
| 38 | TP53_24 | 3608 | 2174 | 2100 | 2111 | 2921 | 4042 |
| 39 | TP53_25 | 3839 | 1825 | 2354 | 2806 | 3415 | 4056 |
| 40 | TP53_26 | 1827 | 783 | 832 | 1106 | 1589 | 1894 |
| 41 | TP53_27 | 363 | 174 | 200 | 340 | 233 | 315 |
| 42 | TP53_28 | 1583 | 764 | 483 | 970 | 1211 | 1516 |
| 43 | TP53_29 | 205 | 71 | 146 | 173 | 218 | 167 |
| 44 | TP53_30 | 132 | 103 | 73 | 86 | 87 | 170 |
| 45 | TP53_31 | 2135 | 1375 | 990 | 1173 | 1782 | 2214 |
| 46 | TP53_32 | 9280 | 4726 | 4931 | 5658 | 7803 | 9325 |
| 47 | TP53_33 | 2789 | 1484 | 1697 | 2281 | 2405 | 3114 |
| 48 | TP53_34 | 4010 | 2739 | 2369 | 2569 | 3329 | 4002 |
| 49 | TP53_35 | 3769 | 3959 | 2382 | 2296 | 2840 | 3939 |
| 50 | TP53_36 | 7524 | 4305 | 3585 | 4532 | 6240 | 7232 |
| 51 | TP53_37 | 10013 | 6100 | 5683 | 6664 | 9067 | 10339 |
| 52 | TP53_38 | 159 | 207 | 120 | 98 | 134 | 212 |
| 53 | TP53_39 | 4783 | 3324 | 3059 | 4490 | 3967 | 5230 |

| **amplicon** | **GENE** | **P36** | **P37** | **P38** | **P39** | **P40** | **P41** |
| --- | --- | --- | --- | --- | --- | --- | --- |
| 1 | BRAF1 | 1393 | 2689 | 3186 | 2695 | 2836 | 3191 |
| 2 | BRAF2 | 5552 | 8991 | 11483 | 11249 | 9208 | 9608 |
| 3 | NRAS1 | 5971 | 5247 | 6763 | 4939 | 6194 | 5843 |
| 4 | NRAS2 | 5205 | 5482 | 6124 | 2400 | 5920 | 6494 |
| 5 | NRAS3 | 4779 | 4730 | 5444 | 2454 | 6223 | 5501 |
| 6 | NRAS4 | 2184 | 2216 | 58 | 1 | 108 | 103 |
| 7 | NRAS5 | 3696 | 4445 | 4473 | 1998 | 4452 | 4344 |
| 8 | KRAS1 | 2988 | 4179 | 6414 | 4149 | 6125 | 6107 |
| 9 | KRAS2 | 1540 | 2260 | 2954 | 1833 | 3859 | 3394 |
| 10 | KRAS3 | 2585 | 2941 | 1898 | 2930 | 4006 | 4224 |
| 11 | KRAS4 | 2826 | 3597 | 5194 | 3626 | 4165 | 4782 |
| 12 | KRAS5 | 3273 | 3122 | 4247 | 3015 | 4010 | 3953 |
| 13 | KRAS6 | 4240 | 4777 | 6719 | 2016 | 5881 | 5572 |
| 14 | KRAS7 | 3110 | 3225 | 4448 | 3541 | 5369 | 5470 |
| 15 | TP53_1 | 9780 | 8798 | 13466 | 9177 | 6529 | 7698 |
| 16 | TP53_2 | 2697 | 2507 | 3956 | 3220 | 2994 | 2896 |
| 17 | TP53_3 | 2264 | 1632 | 2958 | 905 | 1663 | 1644 |
| 18 | TP53_4 | 2689 | 2389 | 2844 | 2049 | 3362 | 3369 |
| 19 | TP53_5 | 2349 | 2072 | 576 | 1818 | 2913 | 2630 |
| 20 | TP53_6 | 2111 | 1936 | 2136 | 2334 | 3012 | 2961 |
| 21 | TP53_7 | 3281 | 2816 | 2854 | 2991 | 4559 | 3802 |
| 22 | TP53_8 | 170 | 154 | 137 | 234 | 199 | 180 |
| 23 | TP53_9 | 4253 | 3683 | 4817 | 3510 | 5130 | 4717 |
| 24 | TP53_10 | 3671 | 3352 | 4486 | 3318 | 3505 | 3608 |
| 25 | TP53_11 | 7044 | 6285 | 7692 | 7783 | 9105 | 8740 |
| 26 | TP53_12 | 544 | 437 | 599 | 563 | 591 | 521 |
| 27 | TP53_13 | 6377 | 6620 | 8819 | 6430 | 7410 | 7581 |
| 28 | TP53_14 | 16427 | 14089 | 20554 | 15355 | 14185 | 14558 |
| 29 | TP53_15 | 11194 | 9517 | 12607 | 9340 | 11241 | 10900 |
| 30 | TP53_16 | 432 | 363 | 320 | 174 | 681 | 659 |
| 31 | TP53_17 | 9914 | 8257 | 12461 | 9958 | 8285 | 8642 |
| 32 | TP53_18 | 4430 | 3687 | 4396 | 3216 | 3858 | 2244 |
| 33 | TP53_19 | 10781 | 8056 | 12484 | 9226 | 6918 | 7438 |
| 34 | TP53_20 | 7450 | 5860 | 8819 | 8117 | 5582 | 6766 |
| 35 | TP53_21 | 6381 | 4908 | 6199 | 5115 | 4610 | 5451 |
| 36 | TP53_22 | 265 | 140 | 129 | 161 | 251 | 275 |
| 37 | TP53_23 | 6829 | 6558 | 9886 | 6514 | 8751 | 8726 |
| 38 | TP53_24 | 3431 | 3524 | 5096 | 3824 | 4223 | 4207 |
| 39 | TP53_25 | 5146 | 3999 | 5646 | 4057 | 5007 | 4673 |
| 40 | TP53_26 | 2492 | 1875 | 2169 | 1762 | 2829 | 2395 |
| 41 | TP53_27 | 243 | 297 | 599 | 201 | 879 | 727 |
| 42 | TP53_28 | 1911 | 1380 | 1992 | 1582 | 2249 | 1907 |
| 43 | TP53_29 | 451 | 262 | 234 | 219 | 319 | 276 |
| 44 | TP53_30 | 330 | 133 | 124 | 231 | 113 | 134 |
| 45 | TP53_31 | 2009 | 2116 | 2737 | 2300 | 2373 | 2645 |
| 46 | TP53_32 | 10083 | 9020 | 11543 | 9263 | 10700 | 11013 |
| 47 | TP53_33 | 3492 | 3127 | 3444 | 2814 | 4694 | 4138 |
| 48 | TP53_34 | 4386 | 3901 | 5575 | 4192 | 4635 | 4584 |
| 49 | TP53_35 | 4306 | 3284 | 5723 | 4572 | 3197 | 3326 |
| 50 | TP53_36 | 8188 | 6298 | 9338 | 7546 | 7488 | 7475 |
| 51 | TP53_37 | 12429 | 10769 | 14009 | 11446 | 12086 | 12130 |
| 52 | TP53_38 | 260 | 188 | 292 | 154 | 382 | 310 |
| 53 | TP53_39 | 5826 | 4872 | 7664 | 5697 | 5235 | 5407 |

| **amplicon** | **GENE** | **P42** | **P43** | **P44** | **P45** | **P46** | **P47** |
| --- | --- | --- | --- | --- | --- | --- | --- |
| 1 | BRAF1 | 2545 | 2932 | 2759 | 3094 | 2676 | 3885 |
| 2 | BRAF2 | 9732 | 11090 | 9953 | 11899 | 10911 | 12190 |
| 3 | NRAS1 | 5597 | 6586 | 5882 | 7085 | 6479 | 6706 |
| 4 | NRAS2 | 5866 | 6431 | 5449 | 7059 | 6120 | 6614 |
| 5 | NRAS3 | 6174 | 6568 | 5596 | 7224 | 6511 | 5448 |
| 6 | NRAS4 | 2530 | 211 | 2690 | 2767 | 20 | 3164 |
| 7 | NRAS5 | 4643 | 4746 | 4289 | 5082 | 4741 | 5221 |
| 8 | KRAS1 | 5840 | 6394 | 5195 | 7016 | 7080 | 5547 |
| 9 | KRAS2 | 2886 | 3305 | 3022 | 4554 | 3512 | 2412 |
| 10 | KRAS3 | 3750 | 4504 | 3631 | 4937 | 4593 | 2867 |
| 11 | KRAS4 | 4428 | 4672 | 4126 | 5625 | 5091 | 6638 |
| 12 | KRAS5 | 3694 | 4022 | 3797 | 4476 | 4722 | 3865 |
| 13 | KRAS6 | 5536 | 5983 | 5601 | 6953 | 7094 | 6530 |
| 14 | KRAS7 | 5007 | 5760 | 4734 | 7171 | 5848 | 4564 |
| 15 | TP53_1 | 8496 | 9135 | 9454 | 9359 | 8376 | 10000 |
| 16 | TP53_2 | 3320 | 3399 | 3185 | 3537 | 2943 | 3004 |
| 17 | TP53_3 | 1244 | 1108 | 1515 | 1025 | 993 | 760 |
| 18 | TP53_4 | 3220 | 2947 | 3020 | 3205 | 2789 | 2563 |
| 19 | TP53_5 | 2023 | 2488 | 2725 | 3090 | 2284 | 1534 |
| 20 | TP53_6 | 2808 | 3264 | 2448 | 2059 | 3007 | 2764 |
| 21 | TP53_7 | 3473 | 3875 | 3576 | 4003 | 3728 | 2991 |
| 22 | TP53_8 | 186 | 299 | 204 | 104 | 208 | 234 |
| 23 | TP53_9 | 4215 | 4854 | 4636 | 4748 | 4436 | 5369 |
| 24 | TP53_10 | 3462 | 3788 | 3732 | 3855 | 3092 | 4022 |
| 25 | TP53_11 | 8466 | 9954 | 9681 | 12100 | 8818 | 7906 |
| 26 | TP53_12 | 458 | 535 | 474 | 564 | 470 | 710 |
| 27 | TP53_13 | 6580 | 7482 | 8005 | 7935 | 6892 | 8771 |
| 28 | TP53_14 | 13785 | 16348 | 15918 | 16702 | 14524 | 14597 |
| 29 | TP53_15 | 10966 | 12716 | 12457 | 11663 | 10956 | 10597 |
| 30 | TP53_16 | 432 | 450 | 466 | 535 | 388 | 164 |
| 31 | TP53_17 | 8228 | 9687 | 9400 | 8178 | 9348 | 11830 |
| 32 | TP53_18 | 3669 | 4626 | 2948 | 4688 | 4221 | 3564 |
| 33 | TP53_19 | 7758 | 9249 | 9956 | 8118 | 9289 | 11251 |
| 34 | TP53_20 | 6079 | 7295 | 7489 | 6589 | 7230 | 8899 |
| 35 | TP53_21 | 4768 | 5410 | 5860 | 5459 | 5471 | 5276 |
| 36 | TP53_22 | 212 | 171 | 254 | 227 | 165 | 155 |
| 37 | TP53_23 | 8548 | 9397 | 8838 | 5931 | 8407 | 9114 |
| 38 | TP53_24 | 4578 | 5485 | 4338 | 4853 | 4253 | 4715 |
| 39 | TP53_25 | 4442 | 5051 | 5312 | 5712 | 4593 | 6327 |
| 40 | TP53_26 | 2205 | 2419 | 2524 | 2556 | 2465 | 1034 |
| 41 | TP53_27 | 684 | 592 | 537 | 747 | 472 | 574 |
| 42 | TP53_28 | 1945 | 2204 | 2164 | 2455 | 1939 | 1229 |
| 43 | TP53_29 | 182 | 162 | 260 | 132 | 201 | 171 |
| 44 | TP53_30 | 112 | 117 | 238 | 52 | 125 | 198 |
| 45 | TP53_31 | 2596 | 2774 | 2567 | 2725 | 2432 | 3283 |
| 46 | TP53_32 | 10242 | 12063 | 11535 | 12412 | 10629 | 12259 |
| 47 | TP53_33 | 3438 | 3630 | 3968 | 4053 | 3472 | 3534 |
| 48 | TP53_34 | 4688 | 5510 | 5093 | 5801 | 4603 | 6872 |
| 49 | TP53_35 | 3827 | 4552 | 4323 | 4463 | 3770 | 4936 |
| 50 | TP53_36 | 7652 | 8703 | 9315 | 9712 | 8524 | 10839 |
| 51 | TP53_37 | 12062 | 13886 | 13531 | 13587 | 12236 | 12123 |
| 52 | TP53_38 | 233 | 245 | 233 | 368 | 175 | 229 |
| 53 | TP53_39 | 5243 | 6119 | 5712 | 5948 | 5772 | 9223 |

| **amplicon** | **GENE** | **P48** | **P49** | **P50** | **P51** | **P52** | **P53** |
| --- | --- | --- | --- | --- | --- | --- | --- |
| 1 | BRAF1 | 4132 | 3891 | 2067 | 1911 | 2396 | 4286 |
| 2 | BRAF2 | 9897 | 10085 | 5953 | 5185 | 5556 | 11716 |
| 3 | NRAS1 | 5803 | 4472 | 3774 | 3651 | 3303 | 6708 |
| 4 | NRAS2 | 6481 | 4891 | 3441 | 3226 | 2918 | 8378 |
| 5 | NRAS3 | 5392 | 3640 | 3349 | 3242 | 2819 | 7216 |
| 6 | NRAS4 | 2762 | 455 | 1727 | 415 | 1615 | 3814 |
| 7 | NRAS5 | 4834 | 3912 | 2550 | 3052 | 2399 | 6373 |
| 8 | KRAS1 | 5371 | 4576 | 3612 | 4045 | 3257 | 5977 |
| 9 | KRAS2 | 3113 | 2412 | 1668 | 1706 | 1939 | 2561 |
| 10 | KRAS3 | 3375 | 2744 | 2347 | 1412 | 1609 | 3759 |
| 11 | KRAS4 | 4165 | 4001 | 2957 | 3049 | 2612 | 5480 |
| 12 | KRAS5 | 3802 | 3402 | 2678 | 2880 | 2375 | 4182 |
| 13 | KRAS6 | 5149 | 4358 | 3225 | 4069 | 2870 | 5413 |
| 14 | KRAS7 | 4481 | 3777 | 2774 | 3299 | 2412 | 4835 |
| 15 | TP53_1 | 6400 | 4050 | 4510 | 3339 | 4368 | 9530 |
| 16 | TP53_2 | 2792 | 1856 | 2297 | 1504 | 2077 | 3788 |
| 17 | TP53_3 | 1036 | 826 | 1531 | 658 | 828 | 1383 |
| 18 | TP53_4 | 2385 | 1553 | 1896 | 1180 | 1706 | 3093 |
| 19 | TP53_5 | 2299 | 1344 | 1402 | 815 | 1552 | 1949 |
| 20 | TP53_6 | 2289 | 1244 | 1312 | 975 | 1440 | 2972 |
| 21 | TP53_7 | 2415 | 1720 | 1775 | 1415 | 1927 | 3613 |
| 22 | TP53_8 | 165 | 140 | 157 | 124 | 141 | 324 |
| 23 | TP53_9 | 4917 | 3191 | 3290 | 2640 | 3404 | 6238 |
| 24 | TP53_10 | 3327 | 1945 | 2272 | 1683 | 2184 | 5096 |
| 25 | TP53_11 | 7322 | 3790 | 3988 | 3098 | 4564 | 8803 |
| 26 | TP53_12 | 574 | 308 | 307 | 202 | 261 | 655 |
| 27 | TP53_13 | 8438 | 4171 | 5027 | 4020 | 4714 | 9480 |
| 28 | TP53_14 | 10657 | 6116 | 6731 | 5213 | 6732 | 15250 |
| 29 | TP53_15 | 9688 | 5981 | 7179 | 4886 | 6938 | 12294 |
| 30 | TP53_16 | 223 | 179 | 155 | 97 | 167 | 262 |
| 31 | TP53_17 | 7990 | 4389 | 5257 | 3818 | 4581 | 10843 |
| 32 | TP53_18 | 3802 | 2389 | 2594 | 1984 | 2945 | 4168 |
| 33 | TP53_19 | 7081 | 4564 | 5364 | 4220 | 5057 | 10032 |
| 34 | TP53_20 | 6097 | 3466 | 4199 | 3552 | 3828 | 8777 |
| 35 | TP53_21 | 4419 | 2767 | 3191 | 2338 | 2982 | 6407 |
| 36 | TP53_22 | 174 | 81 | 103 | 74 | 103 | 214 |
| 37 | TP53_23 | 7985 | 4341 | 4821 | 3375 | 4964 | 11394 |
| 38 | TP53_24 | 3670 | 1987 | 2478 | 1914 | 2478 | 5810 |
| 39 | TP53_25 | 5070 | 2940 | 3641 | 2473 | 3551 | 6191 |
| 40 | TP53_26 | 1258 | 769 | 899 | 581 | 950 | 1273 |
| 41 | TP53_27 | 387 | 224 | 190 | 179 | 210 | 588 |
| 42 | TP53_28 | 1053 | 595 | 635 | 420 | 693 | 1358 |
| 43 | TP53_29 | 231 | 132 | 174 | 161 | 132 | 220 |
| 44 | TP53_30 | 179 | 138 | 147 | 134 | 128 | 217 |
| 45 | TP53_31 | 2171 | 1267 | 1700 | 1240 | 1428 | 3490 |
| 46 | TP53_32 | 9465 | 5584 | 6756 | 4920 | 6492 | 12609 |
| 47 | TP53_33 | 3654 | 2003 | 2227 | 1488 | 2108 | 4018 |
| 48 | TP53_34 | 5113 | 3077 | 3660 | 2611 | 3306 | 7725 |
| 49 | TP53_35 | 3188 | 1719 | 2099 | 1602 | 1973 | 4440 |
| 50 | TP53_36 | 7593 | 4190 | 5571 | 4191 | 5410 | 10522 |
| 51 | TP53_37 | 11131 | 6546 | 7435 | 5227 | 7481 | 12993 |
| 52 | TP53_38 | 115 | 92 | 137 | 75 | 127 | 315 |
| 53 | TP53_39 | 5605 | 3113 | 3723 | 2667 | 3436 | 6802 |

| **amplicon** | **GENE** | **P54** | **P55** | **P56** | **P57** | **P58** | **P59** |
| --- | --- | --- | --- | --- | --- | --- | --- |
| 1 | BRAF1 | 3639 | 3591 | 2107 | 3440 | 3249 | 4076 |
| 2 | BRAF2 | 9768 | 7916 | 4652 | 6528 | 6653 | 7448 |
| 3 | NRAS1 | 4606 | 4601 | 2989 | 4932 | 4618 | 5594 |
| 4 | NRAS2 | 5637 | 4788 | 3077 | 4326 | 4681 | 5768 |
| 5 | NRAS3 | 4344 | 4334 | 2781 | 3889 | 4363 | 5958 |
| 6 | NRAS4 | 378 | 326 | 410 | 2369 | 1935 | 4769 |
| 7 | NRAS5 | 4563 | 3966 | 2542 | 3926 | 3731 | 4630 |
| 8 | KRAS1 | 4035 | 4738 | 3336 | 4334 | 4219 | 6847 |
| 9 | KRAS2 | 1664 | 3041 | 2370 | 3914 | 2106 | 4173 |
| 10 | KRAS3 | 2389 | 3075 | 1969 | 3052 | 2604 | 3943 |
| 11 | KRAS4 | 4533 | 3951 | 2237 | 3576 | 3713 | 4617 |
| 12 | KRAS5 | 2789 | 2569 | 1562 | 2276 | 2633 | 3586 |
| 13 | KRAS6 | 4692 | 4477 | 2966 | 4648 | 3699 | 5392 |
| 14 | KRAS7 | 3319 | 4083 | 2624 | 3677 | 3741 | 5322 |
| 15 | TP53_1 | 8298 | 5665 | 3071 | 5058 | 5458 | 5920 |
| 16 | TP53_2 | 2880 | 2560 | 1822 | 2508 | 2465 | 3081 |
| 17 | TP53_3 | 793 | 803 | 1010 | 1257 | 928 | 2038 |
| 18 | TP53_4 | 2136 | 2103 | 1418 | 2691 | 1994 | 2615 |
| 19 | TP53_5 | 1168 | 1949 | 1651 | 2634 | 1716 | 3028 |
| 20 | TP53_6 | 2438 | 1819 | 1222 | 1709 | 1713 | 2114 |
| 21 | TP53_7 | 2476 | 2416 | 1580 | 2668 | 2139 | 2505 |
| 22 | TP53_8 | 231 | 143 | 139 | 145 | 132 | 128 |
| 23 | TP53_9 | 4071 | 4523 | 3202 | 4594 | 3978 | 5622 |
| 24 | TP53_10 | 3897 | 2852 | 1709 | 2521 | 2933 | 3163 |
| 25 | TP53_11 | 5746 | 7105 | 4062 | 5938 | 5714 | 6833 |
| 26 | TP53_12 | 583 | 426 | 270 | 382 | 447 | 489 |
| 27 | TP53_13 | 7532 | 6876 | 4314 | 6145 | 6133 | 7424 |
| 28 | TP53_14 | 13032 | 11117 | 5856 | 9608 | 9896 | 11467 |
| 29 | TP53_15 | 8004 | 9416 | 5840 | 8743 | 8056 | 10200 |
| 30 | TP53_16 | 212 | 182 | 206 | 537 | 216 | 264 |
| 31 | TP53_17 | 9795 | 6596 | 3568 | 5177 | 5946 | 6258 |
| 32 | TP53_18 | 2550 | 3419 | 1967 | 3311 | 2757 | 4641 |
| 33 | TP53_19 | 9437 | 5832 | 3369 | 4959 | 5958 | 6281 |
| 34 | TP53_20 | 7618 | 4497 | 2890 | 3711 | 4766 | 5271 |
| 35 | TP53_21 | 4560 | 3001 | 2292 | 2661 | 3183 | 4022 |
| 36 | TP53_22 | 119 | 145 | 110 | 228 | 124 | 184 |
| 37 | TP53_23 | 8550 | 5894 | 4130 | 6067 | 6137 | 6717 |
| 38 | TP53_24 | 4232 | 2865 | 1687 | 2475 | 3081 | 3752 |
| 39 | TP53_25 | 4696 | 4398 | 2736 | 4319 | 3921 | 4718 |
| 40 | TP53_26 | 733 | 1317 | 1226 | 1843 | 1145 | 1988 |
| 41 | TP53_27 | 181 | 419 | 316 | 672 | 403 | 525 |
| 42 | TP53_28 | 880 | 1003 | 767 | 1256 | 897 | 1295 |
| 43 | TP53_29 | 139 | 138 | 130 | 165 | 108 | 177 |
| 44 | TP53_30 | 237 | 104 | 73 | 81 | 87 | 135 |
| 45 | TP53_31 | 2528 | 1980 | 1495 | 1994 | 2244 | 2091 |
| 46 | TP53_32 | 9472 | 9203 | 5718 | 8732 | 8118 | 9667 |
| 47 | TP53_33 | 2790 | 3407 | 2561 | 4312 | 2768 | 3935 |
| 48 | TP53_34 | 5774 | 4372 | 2498 | 3918 | 4147 | 4964 |
| 49 | TP53_35 | 4834 | 2798 | 1541 | 2077 | 2530 | 2582 |
| 50 | TP53_36 | 9022 | 6552 | 3821 | 5061 | 6253 | 7571 |
| 51 | TP53_37 | 9018 | 9880 | 6221 | 8624 | 8467 | 10423 |
| 52 | TP53_38 | 247 | 125 | 110 | 186 | 156 | 150 |
| 53 | TP53_39 | 7835 | 4249 | 2321 | 3471 | 3980 | 4153 |

| **amplicon** | **GENE** | **P60** | **P61** | **P62** | **P63** | **P64** | **P65** |
| --- | --- | --- | --- | --- | --- | --- | --- |
| 1 | BRAF1 | 3376 | 2691 | 3701 | 2229 | 3520 | 2239 |
| 2 | BRAF2 | 6556 | 5718 | 6382 | 7186 | 7488 | 5604 |
| 3 | NRAS1 | 4723 | 3978 | 5148 | 4293 | 4909 | 3426 |
| 4 | NRAS2 | 5360 | 3705 | 4368 | 4280 | 4663 | 2984 |
| 5 | NRAS3 | 4326 | 3676 | 4817 | 3629 | 4617 | 2727 |
| 6 | NRAS4 | 4568 | 554 | 3962 | 224 | 4833 | 427 |
| 7 | NRAS5 | 4219 | 3174 | 3647 | 3427 | 3890 | 2933 |
| 8 | KRAS1 | 5273 | 4060 | 5277 | 4491 | 5548 | 3704 |
| 9 | KRAS2 | 4297 | 3128 | 3177 | 3071 | 4227 | 2575 |
| 10 | KRAS3 | 3253 | 2511 | 3023 | 2993 | 3536 | 2526 |
| 11 | KRAS4 | 4874 | 3163 | 3631 | 3602 | 3659 | 2605 |
| 12 | KRAS5 | 3757 | 2336 | 2794 | 2793 | 3712 | 2143 |
| 13 | KRAS6 | 4705 | 3912 | 4368 | 3800 | 4664 | 3191 |
| 14 | KRAS7 | 4242 | 3372 | 3751 | 3576 | 4219 | 2953 |
| 15 | TP53_1 | 5975 | 3912 | 5022 | 4689 | 4628 | 3570 |
| 16 | TP53_2 | 2835 | 1786 | 2588 | 1787 | 2685 | 1760 |
| 17 | TP53_3 | 933 | 1424 | 2095 | 1143 | 2235 | 826 |
| 18 | TP53_4 | 2175 | 1855 | 2369 | 1894 | 2803 | 1680 |
| 19 | TP53_5 | 2722 | 2130 | 2570 | 1947 | 3172 | 1871 |
| 20 | TP53_6 | 2052 | 1106 | 1768 | 1177 | 1820 | 1239 |
| 21 | TP53_7 | 3063 | 1765 | 2340 | 1537 | 2552 | 1979 |
| 22 | TP53_8 | 81 | 68 | 91 | 48 | 111 | 83 |
| 23 | TP53_9 | 6227 | 3720 | 4772 | 3728 | 5282 | 3420 |
| 24 | TP53_10 | 3115 | 1767 | 2212 | 2198 | 2671 | 1746 |
| 25 | TP53_11 | 7108 | 5082 | 5850 | 6192 | 6691 | 4216 |
| 26 | TP53_12 | 466 | 337 | 440 | 389 | 505 | 304 |
| 27 | TP53_13 | 7827 | 4710 | 6606 | 5670 | 6795 | 4056 |
| 28 | TP53_14 | 10314 | 7232 | 9330 | 9026 | 9382 | 6332 |
| 29 | TP53_15 | 9348 | 6272 | 8035 | 6653 | 8386 | 5932 |
| 30 | TP53_16 | 337 | 258 | 263 | 242 | 368 | 213 |
| 31 | TP53_17 | 5697 | 4083 | 5620 | 3865 | 5304 | 3387 |
| 32 | TP53_18 | 4348 | 2949 | 4007 | 3221 | 3653 | 2388 |
| 33 | TP53_19 | 5724 | 4073 | 5290 | 4371 | 5131 | 3564 |
| 34 | TP53_20 | 4916 | 3064 | 4292 | 3566 | 4174 | 2774 |
| 35 | TP53_21 | 3677 | 2398 | 3156 | 2731 | 3295 | 2256 |
| 36 | TP53_22 | 238 | 139 | 176 | 122 | 228 | 124 |
| 37 | TP53_23 | 5412 | 3778 | 6171 | 3503 | 6021 | 4515 |
| 38 | TP53_24 | 2581 | 2160 | 2964 | 1823 | 2572 | 1941 |
| 39 | TP53_25 | 4673 | 3437 | 4336 | 3847 | 4877 | 3235 |
| 40 | TP53_26 | 2008 | 1561 | 1773 | 1079 | 2297 | 1282 |
| 41 | TP53_27 | 452 | 336 | 471 | 238 | 591 | 373 |
| 42 | TP53_28 | 1183 | 978 | 998 | 898 | 1382 | 779 |
| 43 | TP53_29 | 168 | 99 | 162 | 85 | 161 | 135 |
| 44 | TP53_30 | 111 | 60 | 95 | 81 | 75 | 80 |
| 45 | TP53_31 | 1952 | 1300 | 1639 | 1263 | 1844 | 1296 |
| 46 | TP53_32 | 9886 | 6329 | 7000 | 7781 | 8226 | 5631 |
| 47 | TP53_33 | 4375 | 3003 | 3691 | 2767 | 4108 | 2575 |
| 48 | TP53_34 | 4369 | 3200 | 3848 | 3271 | 3898 | 2691 |
| 49 | TP53_35 | 2155 | 1633 | 2067 | 1944 | 2173 | 1664 |
| 50 | TP53_36 | 7229 | 4477 | 6151 | 5488 | 5718 | 3783 |
| 51 | TP53_37 | 10600 | 6308 | 8148 | 7558 | 8471 | 6374 |
| 52 | TP53_38 | 141 | 82 | 116 | 71 | 137 | 107 |
| 53 | TP53_39 | 4097 | 2302 | 3551 | 3818 | 4158 | 2803 |

| **amplicon** | **GENE** | **P66** | **P67** | **P68** | **P69** | **P70** | **P71** |
| --- | --- | --- | --- | --- | --- | --- | --- |
| 1 | BRAF1 | 3654 | 2363 | 4197 | 4871 | 3542 | 2142 |
| 2 | BRAF2 | 8571 | 5777 | 9440 | 12545 | 8873 | 8256 |
| 3 | NRAS1 | 5766 | 4052 | 6015 | 8608 | 5347 | 4921 |
| 4 | NRAS2 | 4782 | 3605 | 6005 | 7611 | 5011 | 5393 |
| 5 | NRAS3 | 4959 | 3182 | 5339 | 6958 | 4501 | 3980 |
| 6 | NRAS4 | 968 | 2078 | 5675 | 4427 | 357 | 3101 |
| 7 | NRAS5 | 4825 | 3332 | 5223 | 6738 | 4378 | 4063 |
| 8 | KRAS1 | 6149 | 4448 | 7669 | 9233 | 5745 | 4488 |
| 9 | KRAS2 | 4086 | 3241 | 4959 | 7574 | 3234 | 2631 |
| 10 | KRAS3 | 4018 | 2723 | 4613 | 6332 | 3714 | 2852 |
| 11 | KRAS4 | 4348 | 3114 | 5153 | 7196 | 4007 | 3664 |
| 12 | KRAS5 | 4129 | 2889 | 4535 | 5752 | 3411 | 3032 |
| 13 | KRAS6 | 5561 | 1773 | 6244 | 8749 | 4473 | 5001 |
| 14 | KRAS7 | 4641 | 3353 | 5696 | 7572 | 4427 | 3334 |
| 15 | TP53_1 | 5951 | 4191 | 6991 | 8359 | 5660 | 6969 |
| 16 | TP53_2 | 3372 | 2136 | 3548 | 3891 | 2688 | 2593 |
| 17 | TP53_3 | 1423 | 595 | 1503 | 1371 | 1871 | 1290 |
| 18 | TP53_4 | 2808 | 1998 | 3007 | 3973 | 2484 | 2001 |
| 19 | TP53_5 | 2913 | 2114 | 2878 | 4442 | 1921 | 1846 |
| 20 | TP53_6 | 2421 | 1355 | 2543 | 2438 | 1781 | 1086 |
| 21 | TP53_7 | 3287 | 2172 | 3348 | 4529 | 1923 | 2089 |
| 22 | TP53_8 | 199 | 44 | 192 | 159 | 106 | 85 |
| 23 | TP53_9 | 5905 | 3964 | 6514 | 8317 | 5006 | 2807 |
| 24 | TP53_10 | 3123 | 2078 | 3756 | 3830 | 2704 | 3070 |
| 25 | TP53_11 | 6811 | 5185 | 7928 | 10476 | 6667 | 7480 |
| 26 | TP53_12 | 452 | 388 | 436 | 671 | 401 | 333 |
| 27 | TP53_13 | 7685 | 5295 | 8956 | 9760 | 7165 | 5533 |
| 28 | TP53_14 | 10822 | 7794 | 13060 | 14797 | 10451 | 13021 |
| 29 | TP53_15 | 9884 | 6251 | 11478 | 12614 | 9047 | 7879 |
| 30 | TP53_16 | 422 | 213 | 387 | 649 | 191 | 237 |
| 31 | TP53_17 | 5799 | 3937 | 6359 | 7645 | 5548 | 6069 |
| 32 | TP53_18 | 3795 | 3211 | 4292 | 5372 | 3604 | 1841 |
| 33 | TP53_19 | 5860 | 4077 | 6782 | 7760 | 5735 | 6449 |
| 34 | TP53_20 | 4881 | 3220 | 5598 | 6572 | 4629 | 5579 |
| 35 | TP53_21 | 3716 | 2868 | 4279 | 5229 | 3516 | 4337 |
| 36 | TP53_22 | 236 | 123 | 235 | 317 | 175 | 128 |
| 37 | TP53_23 | 7382 | 3664 | 7770 | 8137 | 6321 | 4371 |
| 38 | TP53_24 | 3016 | 2116 | 3427 | 3690 | 2612 | 2612 |
| 39 | TP53_25 | 5002 | 3332 | 5620 | 7354 | 4798 | 4076 |
| 40 | TP53_26 | 2127 | 1561 | 2298 | 3892 | 1505 | 1357 |
| 41 | TP53_27 | 570 | 464 | 577 | 904 | 449 | 309 |
| 42 | TP53_28 | 1279 | 858 | 1515 | 2193 | 1067 | 1550 |
| 43 | TP53_29 | 154 | 129 | 209 | 229 | 150 | 122 |
| 44 | TP53_30 | 116 | 52 | 129 | 115 | 109 | 42 |
| 45 | TP53_31 | 2017 | 1233 | 2192 | 2865 | 1698 | 2254 |
| 46 | TP53_32 | 9627 | 6966 | 11253 | 13314 | 8609 | 9356 |
| 47 | TP53_33 | 4373 | 2773 | 4770 | 6573 | 3412 | 2427 |
| 48 | TP53_34 | 4725 | 3240 | 5295 | 6539 | 4029 | 3730 |
| 49 | TP53_35 | 2698 | 1937 | 2983 | 3372 | 2312 | 4018 |
| 50 | TP53_36 | 6927 | 5134 | 7951 | 9138 | 6613 | 6765 |
| 51 | TP53_37 | 10489 | 7202 | 12442 | 14085 | 9268 | 9101 |
| 52 | TP53_38 | 172 | 114 | 242 | 288 | 113 | 211 |
| 53 | TP53_39 | 4664 | 3318 | 6256 | 6000 | 4559 | 5086 |

| **amplicon** | **GENE** | **P72** | **P73** | **P74** | **P75** | **P76** | **P77** |
| --- | --- | --- | --- | --- | --- | --- | --- |
| 1 | BRAF1 | 1671 | 2874 | 4541 | 5496 | 2243 | 1986 |
| 2 | BRAF2 | 4113 | 8578 | 12698 | 16284 | 6032 | 5063 |
| 3 | NRAS1 | 2514 | 4814 | 4626 | 8224 | 3556 | 2746 |
| 4 | NRAS2 | 2794 | 5559 | 6453 | 8646 | 3698 | 3536 |
| 5 | NRAS3 | 2179 | 3869 | 5044 | 5808 | 2956 | 2368 |
| 6 | NRAS4 | 918 | 3235 | 2525 | 3000 | 2107 | 1919 |
| 7 | NRAS5 | 2482 | 4507 | 6111 | 7648 | 3343 | 2774 |
| 8 | KRAS1 | 2236 | 4359 | 5852 | 8327 | 3215 | 2696 |
| 9 | KRAS2 | 1576 | 2618 | 3804 | 4586 | 2089 | 1593 |
| 10 | KRAS3 | 1806 | 3250 | 3099 | 5872 | 2297 | 1663 |
| 11 | KRAS4 | 1687 | 4005 | 5371 | 8184 | 2654 | 1903 |
| 12 | KRAS5 | 2050 | 3075 | 4685 | 6811 | 2337 | 1812 |
| 13 | KRAS6 | 1552 | 4982 | 6670 | 5726 | 3306 | 2628 |
| 14 | KRAS7 | 1807 | 3623 | 4219 | 5651 | 2352 | 1877 |
| 15 | TP53_1 | 3261 | 8210 | 7532 | 14306 | 5062 | 3722 |
| 16 | TP53_2 | 1583 | 3171 | 2942 | 5463 | 2001 | 1760 |
| 17 | TP53_3 | 463 | 482 | 1531 | 1114 | 1199 | 645 |
| 18 | TP53_4 | 1125 | 2088 | 1784 | 2831 | 1766 | 1361 |
| 19 | TP53_5 | 962 | 1600 | 1534 | 2519 | 1228 | 1194 |
| 20 | TP53_6 | 1160 | 2301 | 2298 | 2514 | 1394 | 1312 |
| 21 | TP53_7 | 1491 | 3111 | 2593 | 3944 | 2036 | 1891 |
| 22 | TP53_8 | 130 | 132 | 192 | 269 | 124 | 158 |
| 23 | TP53_9 | 1745 | 3417 | 3365 | 6216 | 2693 | 2300 |
| 24 | TP53_10 | 1774 | 3885 | 3327 | 6740 | 2354 | 1924 |
| 25 | TP53_11 | 2932 | 7213 | 5919 | 10528 | 4462 | 3611 |
| 26 | TP53_12 | 163 | 420 | 298 | 831 | 334 | 226 |
| 27 | TP53_13 | 3196 | 6090 | 6115 | 10917 | 4742 | 3947 |
| 28 | TP53_14 | 6115 | 13246 | 12044 | 22215 | 9033 | 7207 |
| 29 | TP53_15 | 4562 | 8738 | 8673 | 14588 | 6506 | 5575 |
| 30 | TP53_16 | 144 | 241 | 334 | 387 | 206 | 308 |
| 31 | TP53_17 | 3735 | 8006 | 7140 | 14808 | 5641 | 4766 |
| 32 | TP53_18 | 1485 | 2868 | 2950 | 2412 | 2423 | 1556 |
| 33 | TP53_19 | 4024 | 7966 | 7647 | 15335 | 5308 | 3819 |
| 34 | TP53_20 | 3216 | 5310 | 5807 | 11693 | 4042 | 2894 |
| 35 | TP53_21 | 2326 | 4324 | 4749 | 8965 | 3434 | 2391 |
| 36 | TP53_22 | 67 | 70 | 110 | 244 | 87 | 88 |
| 37 | TP53_23 | 4277 | 6207 | 6688 | 12807 | 5009 | 4260 |
| 38 | TP53_24 | 1664 | 3766 | 3290 | 5954 | 2140 | 1764 |
| 39 | TP53_25 | 1818 | 4059 | 3832 | 6705 | 2943 | 2246 |
| 40 | TP53_26 | 748 | 1547 | 1851 | 2494 | 1149 | 796 |
| 41 | TP53_27 | 282 | 445 | 419 | 549 | 397 | 326 |
| 42 | TP53_28 | 801 | 1690 | 1363 | 2561 | 1045 | 952 |
| 43 | TP53_29 | 140 | 171 | 152 | 327 | 91 | 159 |
| 44 | TP53_30 | 102 | 91 | 96 | 345 | 60 | 104 |
| 45 | TP53_31 | 1437 | 2637 | 2251 | 4479 | 1687 | 1393 |
| 46 | TP53_32 | 4101 | 9204 | 8737 | 15228 | 6031 | 4988 |
| 47 | TP53_33 | 1677 | 2784 | 2914 | 4711 | 2201 | 1967 |
| 48 | TP53_34 | 1841 | 4525 | 4130 | 7827 | 2865 | 2239 |
| 49 | TP53_35 | 1954 | 4688 | 3980 | 8020 | 2741 | 2298 |
| 50 | TP53_36 | 3213 | 7134 | 6271 | 12751 | 5172 | 3638 |
| 51 | TP53_37 | 4987 | 10251 | 9988 | 17769 | 7410 | 5925 |
| 52 | TP53_38 | 157 | 287 | 199 | 498 | 168 | 121 |
| 53 | TP53_39 | 2512 | 5481 | 5056 | 9305 | 3579 | 3551 |

| **amplicon** | **GENE** | **P78** | **P79** | **P80** | **P81** | **P82** | **P83** |
| --- | --- | --- | --- | --- | --- | --- | --- |
| 1 | BRAF1 | 2298 | 6702 | 2685 | 3147 | 2101 | 2940 |
| 2 | BRAF2 | 5527 | 18009 | 7135 | 7637 | 5313 | 8530 |
| 3 | NRAS1 | 3498 | 7420 | 4182 | 4559 | 2913 | 5105 |
| 4 | NRAS2 | 4346 | 7816 | 4736 | 5440 | 3131 | 5727 |
| 5 | NRAS3 | 2747 | 5901 | 4246 | 3981 | 2595 | 4336 |
| 6 | NRAS4 | 1118 | 2859 | 1481 | 1413 | 2673 | 2417 |
| 7 | NRAS5 | 3011 | 6463 | 3913 | 4148 | 2621 | 4279 |
| 8 | KRAS1 | 3778 | 6983 | 4557 | 4677 | 3181 | 5014 |
| 9 | KRAS2 | 2687 | 3979 | 2608 | 2944 | 1874 | 3527 |
| 10 | KRAS3 | 2347 | 2412 | 2679 | 3158 | 2205 | 3017 |
| 11 | KRAS4 | 2834 | 5705 | 3601 | 3470 | 2130 | 4305 |
| 12 | KRAS5 | 2183 | 4619 | 2941 | 2868 | 1959 | 3175 |
| 13 | KRAS6 | 3716 | 7226 | 4404 | 4536 | 2799 | 5203 |
| 14 | KRAS7 | 3026 | 4651 | 3469 | 3226 | 2184 | 3102 |
| 15 | TP53_1 | 3936 | 5379 | 6174 | 6217 | 4064 | 5893 |
| 16 | TP53_2 | 1642 | 2243 | 2307 | 2354 | 1749 | 1984 |
| 17 | TP53_3 | 532 | 729 | 516 | 768 | 1327 | 461 |
| 18 | TP53_4 | 1487 | 1659 | 1729 | 1970 | 1609 | 2110 |
| 19 | TP53_5 | 1544 | 1657 | 1628 | 2008 | 1329 | 1894 |
| 20 | TP53_6 | 1502 | 1839 | 2000 | 1989 | 1309 | 665 |
| 21 | TP53_7 | 1996 | 2561 | 2820 | 3100 | 1987 | 1701 |
| 22 | TP53_8 | 62 | 172 | 154 | 204 | 131 | 27 |
| 23 | TP53_9 | 2651 | 3066 | 3383 | 3504 | 2324 | 2598 |
| 24 | TP53_10 | 2042 | 2919 | 3437 | 3153 | 2117 | 3202 |
| 25 | TP53_11 | 4281 | 5545 | 6147 | 5991 | 3914 | 8667 |
| 26 | TP53_12 | 285 | 314 | 351 | 501 | 259 | 330 |
| 27 | TP53_13 | 4073 | 5019 | 5518 | 5913 | 4170 | 7138 |
| 28 | TP53_14 | 7493 | 9885 | 12111 | 11411 | 7937 | 13917 |
| 29 | TP53_15 | 5136 | 7327 | 8284 | 8067 | 5980 | 6668 |
| 30 | TP53_16 | 375 | 323 | 221 | 412 | 293 | 382 |
| 31 | TP53_17 | 4499 | 5892 | 7353 | 6889 | 4671 | 4941 |
| 32 | TP53_18 | 2269 | 2188 | 2820 | 2909 | 2327 | 627 |
| 33 | TP53_19 | 3730 | 5150 | 6476 | 5937 | 4080 | 4978 |
| 34 | TP53_20 | 2859 | 4118 | 4573 | 4845 | 3298 | 4251 |
| 35 | TP53_21 | 2857 | 3452 | 3874 | 4931 | 2842 | 4107 |
| 36 | TP53_22 | 85 | 77 | 103 | 146 | 95 | 101 |
| 37 | TP53_23 | 3780 | 5144 | 5591 | 6069 | 4260 | 3374 |
| 38 | TP53_24 | 1933 | 2546 | 3423 | 2960 | 1921 | 2556 |
| 39 | TP53_25 | 2610 | 423 | 3501 | 3486 | 2531 | 4702 |
| 40 | TP53_26 | 1133 | 213 | 1567 | 1598 | 1096 | 1295 |
| 41 | TP53_27 | 457 | 233 | 489 | 303 | 349 | 126 |
| 42 | TP53_28 | 1012 | 1096 | 1402 | 1398 | 1009 | 1280 |
| 43 | TP53_29 | 89 | 57 | 83 | 182 | 101 | 149 |
| 44 | TP53_30 | 37 | 124 | 73 | 150 | 59 | 45 |
| 45 | TP53_31 | 1401 | 255 | 1913 | 2088 | 1401 | 2245 |
| 46 | TP53_32 | 5728 | 1043 | 8087 | 8158 | 5334 | 9412 |
| 47 | TP53_33 | 2517 | 334 | 2603 | 3391 | 2102 | 2106 |
| 48 | TP53_34 | 2692 | 435 | 3740 | 3834 | 2293 | 3610 |
| 49 | TP53_35 | 1962 | 2608 | 3697 | 3299 | 2192 | 3546 |
| 50 | TP53_36 | 3928 | 780 | 5952 | 6000 | 4299 | 5923 |
| 51 | TP53_37 | 5742 | 1072 | 9006 | 8795 | 6117 | 9180 |
| 52 | TP53_38 | 181 | 17 | 275 | 259 | 159 | 197 |
| 53 | TP53_39 | 3313 | 527 | 5237 | 4808 | 3606 | 4508 |
| **amplicon** | **GENE** | **P84** | **P85** | **P86** | **P87** |  |  |
| 1 | BRAF1 | 2173 | 2707 | 4817 | 3676 |  |  |
| 2 | BRAF2 | 5195 | 8243 | 14281 | 9944 |  |  |
| 3 | NRAS1 | 2783 | 4455 | 7531 | 5410 |  |  |
| 4 | NRAS2 | 3567 | 5516 | 9879 | 6430 |  |  |
| 5 | NRAS3 | 2615 | 3906 | 7175 | 4966 |  |  |
| 6 | NRAS4 | 606 | 1202 | 6598 | 3196 |  |  |
| 7 | NRAS5 | 2867 | 4356 | 7765 | 4905 |  |  |
| 8 | KRAS1 | 3036 | 4536 | 9073 | 5613 |  |  |
| 9 | KRAS2 | 2246 | 3001 | 5918 | 3648 |  |  |
| 10 | KRAS3 | 2126 | 3254 | 5859 | 4025 |  |  |
| 11 | KRAS4 | 2314 | 4165 | 7323 | 4891 |  |  |
| 12 | KRAS5 | 2350 | 2904 | 5306 | 4005 |  |  |
| 13 | KRAS6 | 3498 | 5171 | 9224 | 6767 |  |  |
| 14 | KRAS7 | 2423 | 2991 | 5667 | 3754 |  |  |
| 15 | TP53_1 | 3644 | 5558 | 9648 | 7894 |  |  |
| 16 | TP53_2 | 1659 | 2354 | 3760 | 3042 |  |  |
| 17 | TP53_3 | 1096 | 339 | 2324 | 581 |  |  |
| 18 | TP53_4 | 1521 | 1887 | 3263 | 2491 |  |  |
| 19 | TP53_5 | 1456 | 1589 | 3551 | 2178 |  |  |
| 20 | TP53_6 | 1252 | 827 | 1740 | 1492 |  |  |
| 21 | TP53_7 | 1633 | 1926 | 3187 | 2945 |  |  |
| 22 | TP53_8 | 103 | 50 | 124 | 90 |  |  |
| 23 | TP53_9 | 2400 | 2106 | 4085 | 4022 |  |  |
| 24 | TP53_10 | 2136 | 3430 | 5121 | 4566 |  |  |
| 25 | TP53_11 | 4132 | 7852 | 12919 | 9444 |  |  |
| 26 | TP53_12 | 289 | 299 | 498 | 505 |  |  |
| 27 | TP53_13 | 4040 | 6295 | 12045 | 7975 |  |  |
| 28 | TP53_14 | 7436 | 12926 | 19622 | 15927 |  |  |
| 29 | TP53_15 | 5260 | 4905 | 9975 | 8125 |  |  |
| 30 | TP53_16 | 253 | 317 | 733 | 335 |  |  |
| 31 | TP53_17 | 4155 | 4644 | 7482 | 7486 |  |  |
| 32 | TP53_18 | 1819 | 3047 | 5991 | 4059 |  |  |
| 33 | TP53_19 | 3838 | 4663 | 7395 | 7170 |  |  |
| 34 | TP53_20 | 2901 | 3888 | 7681 | 5741 |  |  |
| 35 | TP53_21 | 2523 | 3489 | 6278 | 5166 |  |  |
| 36 | TP53_22 | 77 | 95 | 248 | 117 |  |  |
| 37 | TP53_23 | 3875 | 2545 | 4639 | 5371 |  |  |
| 38 | TP53_24 | 1884 | 2466 | 4514 | 4027 |  |  |
| 39 | TP53_25 | 2361 | 3979 | 7160 | 5168 |  |  |
| 40 | TP53_26 | 1095 | 1093 | 2058 | 1870 |  |  |
| 41 | TP53_27 | 345 | 377 | 710 | 508 |  |  |
| 42 | TP53_28 | 926 | 1191 | 2497 | 1761 |  |  |
| 43 | TP53_29 | 97 | 89 | 211 | 180 |  |  |
| 44 | TP53_30 | 67 | 37 | 72 | 104 |  |  |
| 45 | TP53_31 | 1415 | 2158 | 3321 | 2812 |  |  |
| 46 | TP53_32 | 5336 | 8756 | 14531 | 11285 |  |  |
| 47 | TP53_33 | 2196 | 1820 | 4465 | 3284 |  |  |
| 48 | TP53_34 | 2355 | 3213 | 5551 | 4959 |  |  |
| 49 | TP53_35 | 2283 | 3448 | 4482 | 4651 |  |  |
| 50 | TP53_36 | 3504 | 5272 | 8953 | 7475 |  |  |
| 51 | TP53_37 | 5831 | 9278 | 14361 | 12423 |  |  |
| 52 | TP53_38 | 109 | 176 | 386 | 254 |  |  |
| 53 | TP53_39 | 2601 | 4022 | 6591 | 5093 |  |  |
