# Supplementary material for: Combined assessment of the TNM stage and BRAF mutational status at diagnosis in sporadic colorectal cancer patients
Source: Oncotarget. 2018 May 8;9(35):24081–96. doi: 10.18632/oncotarget.25300 (PMC5963609; doi:10.18632/oncotarget.25300)
Supplement: Supplementary file 3 [file oncotarget-09-24081-s003.docx]

**Supplementary Table 2:** KRAS, BRAF, NRAS and TP53 variants detected and their variant allele frequency (VAF) per tumor/case analyzed, the administered treatment and microsatellite status.

| **PATIENT** | **KRAS variant** | **KRAS allele burden** | **BRAF variant** | **BRAF allele burden** | **NRAS variant** | **NRAS allele burden** | **TP53**  **variant** | **TP53 allele burden** | **Treatment** | **Microsatellite instability** |
| --- | --- | --- | --- | --- | --- | --- | --- | --- | --- | --- |
| Patient 1 | A146P | 44 | Wild-type | 0 | Wild-type | 0 | Wild-type | 0 |  | NO |
| Patient 2 | A146T | 15 | Wild-type | 0 | Wild-type | 0 | Wild-type | 0 | Capecitabine |  |
| Patient 3 | G12A | 34 | Wild-type | 0 | Wild-type | 0 | R273C | 26 |  | NO |
| Patient 4 | G12A | 36 | Wild-type | 0 | Wild-type | 0 | S215R | 38 |  |  |
| Patient 5 | G12A | 36 | Wild-type | 0 | Wild-type | 0 | R175H | 32 | Xelox | NO |
| Patient 6 | G12A | 41 | Wild-type | 0 | Wild-type | 0 | Wild-type | 0 |  | NO |
| Patient 7 | G12D | 2 | Wild-type | 0 | Wild-type | 0 | L111R | 3 |  | NO |
| Patient 8 | G12D | 24 | Wild-type | 0 | Wild-type | 0 | Wild-type | 0 |  |  |
| Patient 9 | G12D | 27 | Wild-type | 0 | Wild-type | 0 | Wild-type | 0 |  | NO |
| Patient 10 | G12D | 39 | Wild-type | 0 | Wild-type | 0 | Wild-type | 0 |  | NO |
| Patient 11 | G12D | 43 | Wild-type | 0 | Wild-type | 0 | Wild-type | 0 | Capecitabine |  |
| Patient 12 | G12D | 45 | Wild-type | 0 | Wild-type | 0 | Wild-type | 0 |  | NO |
| Patient 13 | G12D | 47 | Wild-type | 0 | Wild-type | 0 | Wild-type | 0 | Xelox | NO |
| Patient 14 | G12S | 27 | Wild-type | 0 | Wild-type | 0 | Wild-type | 0 |  |  |
| Patient 15 | G12V | 13 | Wild-type | 0 | Wild-type | 0 | Wild-type | 0 |  | NO |
| Patient 16 | G12V | 24 | Wild-type | 0 | Wild-type | 0 | T253P fsTer92 | 44 |  |  |
| Patient 17 | G12V | 28 | Wild-type | 0 | Wild-type | 0 | Wild-type | 0 |  | NO |
| Patient 18 | G12V | 30 | Wild-type | 0 | Wild-type | 0 | R282W | 30 |  | NO |
| Patient 19 | G12V | 31 | Wild-type | 0 | Wild-type | 0 | R156H | 27 |  | NO |
| Patient 20 | G13D | 10 | Wild-type | 0 | Wild-type | 0 | R248W | 9 |  |  |
| Patient 21 | G13D | 42 | Wild-type | 0 | Wild-type | 0 | Wild-type | 0 | Xelox | NO |
| Patient 22 | K147E | 52 | Wild-type | 0 | Wild-type | 0 | Wild-type | 0 |  |  |
| Patient 23 | Q61H | 41 | Wild-type | 0 | Wild-type | 0 | R175H | 51 | Xelox | NO |
| Patient 24 | Q61L | 23 | Wild-type | 0 | Wild-type | 0 | Cys277ValfsTer68 | 14 | Xelox | NO |
| Patient 25 | Wild-type | 0 | V600E | 40 | Wild-type | 0 | C275Y | 72 |  | NO |
| Patient 26 | Wild-type | 0 | V600E | 30 | Wild-type | 0 | Wild-type | 0 |  | YES |
| Patient 27 | Wild-type | 0 | V600E | 8,5 | Wild-type | 0 | R158C | 9 | Capecitabine | YES |
| Patient 28 | Wild-type | 0 | V600E | 20 | Wild-type | 0 | Wild-type | 0 |  | YES |
| Patient 29 | Wild-type | 0 | V600E | 32 | Wild-type | 0 | R196* | 84 | Xelox |  |
| Patient 30 | Wild-type | 0 | V600E | 6 | Wild-type | 0 | V272M | 1,8 |  | NO |
| Patient 31 | Wild-type | 0 | Wild-type | 0 | Wild-type | 0 | His178ProfsTer3 | 42 | Capecitabine | NO |
| Patient 32 | Wild-type | 0 | Wild-type | 0 | Wild-type | 0 | R282W | 23 | Xelox | NO |
| Patient 33 | Wild-type | 0 | Wild-type | 0 | Wild-type | 0 | Q192* | 85 |  | NO |
| Patient 34 | Wild-type | 0 | Wild-type | 0 | Wild-type | 0 | G112- | 68 |  | NO |
| Patient 35 | Wild-type | 0 | Wild-type | 0 | Wild-type | 0 | Pro152AlafsTer14 | 63 | Xelox | NO |
| Patient 36 | Wild-type | 0 | Wild-type | 0 | Wild-type | 0 | N247I | 74 |  | NO |
| Patient 37 | Wild-type | 0 | Wild-type | 0 | Wild-type | 0 | Leu201GlyfsTer47 | 50 |  |  |
| Patient 38 | Wild-type | 0 | Wild-type | 0 | Wild-type |  | Wild-type | 0 | Tomox |  |
| Patient 39 | Wild-type | 0 | Wild-type | 0 | Wild-type | 0 | c.376-2A>G SPLICE | 54 |  | NO |
| Patient 40 | Wild-type | 0 | Wild-type | 0 | Wild-type | 0 | V173L | 89 | Xelox |  |
| Patient 41 | Wild-type | 0 | Wild-type | 0 | Wild-type | 0 | R248Q | 21 | Tomudex | NO |
| Patient 42 | Wild-type | 0 | Wild-type | 0 | Wild-type | 0 | Wild-type | 0 |  | YES |
| Patient 43 | Wild-type | 0 | Wild-type | 0 | Wild-type | 0 | C135F | 17 |  | NO |
| Patient 44 | Wild-type | 0 | Wild-type | 0 | Wild-type | 0 | Wild-type | 0 | Xelox | NO |
| Patient 45 | Wild-type | 0 | Wild-type | 0 | Wild-type | 0 | Wild-type | 0 | Capecitabine | NO |
| Patient 46 | Wild-type | 0 | Wild-type | 0 | Wild-type | 0 | Wild-type | 0 |  | NO |
| Patient 47 | Wild-type | 0 | Wild-type | 0 | Wild-type | 0 | Wild-type | 0 |  |  |
| Patient 48 | Wild-type | 0 | Wild-type | 0 | Wild-type | 0 | Wild-type | 0 |  | YES |
| Patient 49 | Wild-type | 0 | Wild-type | 0 | Wild-type | 0 | Wild-type | 0 |  | NO |
| Patient 50 | Wild-type | 0 | Wild-type | 0 | Wild-type | 0 | R175H | 1,5 |  | NO |
| Patient 51 | Wild-type | 0 | Wild-type | 0 | Wild-type | 0 | R282W | 48 | Xelox | NO |
| Patient 52 | Wild-type | 0 | Wild-type | 0 | mutado | 2,5 | Wild-type | 0 |  |  |
| Patient 53 | Wild-type | 0 | Wild-type | 0 | Wild-type | 0 | Wild-type | 0 |  | NO |
| Patient 54 | Wild-type | 0 | Wild-type | 0 | Wild-type | 0 | Wild-type | 0 | Xelox | NO |
| Patient 55 | Wild-type | 0 | Wild-type | 0 | Wild-type | 0 | Wild-type | 0 | Capecitabine | NO |
| Patient 56 | Wild-type | 0 | Wild-type | 0 | Wild-type | 0 | Wild-type | 0 | Xelox | NO |
| Patient 57 | Wild-type | 0 | Wild-type | 0 | Wild-type | 0 | Wild-type | 0 |  |  |
| Patient 58 | Wild-type | 0 | Wild-type | 0 | Wild-type | 0 | Wild-type | 0 |  | NO |
| Patient 59 | Wild-type | 0 | Wild-type | 0 | Wild-type | 0 | Wild-type | 0 |  |  |
| Patient 60 | Wild-type | 0 | Wild-type | 0 | Wild-type | 0 | Wild-type | 0 | Capecitabine | NO |
| Patient 61 | Wild-type | 0 | Wild-type | 0 | Wild-type | 0 | Wild-type | 0 |  | NO |
| Patient 62 | Wild-type | 0 | Wild-type | 0 | Wild-type | 0 | Wild-type | 0 |  |  |
| Patient 63 | Wild-type | 0 | Wild-type | 0 | Wild-type | 0 | Wild-type | 0 |  | NO |
| Patient 64 | Wild-type | 0 | Wild-type | 0 | Wild-type | 0 | Wild-type | 0 | Capecitabine |  |
| Patient 65 | Wild-type | 0 | Wild-type | 0 | Wild-type |  | Wild-type | 0 |  |  |
| Patient 66 | Wild-type | 0 | Wild-type | 0 | Wild-type | 0 | Wild-type | 0 |  |  |
| Patient 67 | Wild-type | 0 | Wild-type | 0 | Wild-type | 0 | Wild-type | 0 |  |  |
| Patient 68 | Wild-type | 0 | Wild-type | 0 | Wild-type | 0 | Wild-type | 0 | Xelox | NO |
| Patient 69 | Wild-type | 0 | Wild-type | 0 | Wild-type | 0 | Wild-type | 0 |  | NO |
| Patient 70 | Wild-type | 0 | Wild-type | 0 | Wild-type | 0 | Wild-type | 0 | Xelox/RT |  |
| Patient 71 | Wild-type | 0 | Wild-type | 0 | Wild-type | 0 | Wild-type | 0 | Capecitabine |  |
| Patient 72 | Wild-type | 0 | Wild-type | 0 | Wild-type | 0 | Wild-type | 0 | Capecitabine |  |
| Patient 73 | Wild-type | 0 | Wild-type | 0 | Wild-type |  | Wild-type | 0 |  |  |
| Patient 74 | Wild-type | 0 | Wild-type | 0 | Wild-type | 0 | R248W | 62 | Tomox |  |
| Patient 75 | Wild-type | 0 | Wild-type | 0 | Wild-type | 0 | S366A | 41 | Xelox | NO |
| Patient 76 | Wild-type | 0 | Wild-type | 0 | Wild-type | 0 | Wild-type | 0 | Utefox | NO |
| Patient 77 | Wild-type | 0 | Wild-type | 0 | Wild-type | 0 | Wild-type | 0 |  |  |
| Patient 78 | Wild-type | 0 | Wild-type | 0 | Wild-type | 0 | Wild-type | 0 | Xelox | YES |
| Patient 79 | Wild-type | 0 | Wild-type | 0 | Wild-type | 0 | Wild-type | 0 | Cisplatin/Etoposide |  |
| Patient 80 | Wild-type | 0 | Wild-type | 0 | Wild-type | 0 | Wild-type | 0 |  |  |
| Patient 81 | Wild-type | 0 | Wild-type | 0 | Wild-type | 0 | Wild-type | 0 |  |  |
| Patient 82 | Wild-type | 0 | Wild-type | 0 | Wild-type | 0 | Wild-type | 0 | Capecitabine |  |
| Patient 83 | Wild-type | 0 | Wild-type | 0 | Wild-type | 0 | Wild-type | 0 | Capecitabine | NO |
| Patient 84 | Wild-type | 0 | Wild-type | 0 | Wild-type | 0 | Wild-type | 0 |  |  |
| Patient 85 | Wild-type | 0 | Wild-type | 0 | Wild-type | 0 | Wild-type | 0 |  |  |
| Patient 86 | Wild-type | 0 | Wild-type | 0 | Wild-type | 0 | Wild-type | 0 |  | NO |
| Patient 87 | Wild-type | 0 | Wild-type | 0 | Wild-type | 0 | Wild-type | 0 |  | NO |
